# Modular enzyme assembly for enhanced cascade biocatalysis and metabolic flux

**DOI:** 10.1038/s41467-019-12247-w

**Published:** 2019-09-18

**Authors:** Wei Kang, Tian Ma, Min Liu, Jiale Qu, Zhenjun Liu, Huawei Zhang, Bin Shi, Shuai Fu, Juncai Ma, Louis Tung Faat Lai, Sicong He, Jianan Qu, Shannon Wing-Ngor Au, Byung Ho Kang, Wilson Chun Yu Lau, Zixin Deng, Jiang Xia, Tiangang Liu

**Affiliations:** 1 Department of Chemistry, The Chinese University of Hong Kong, Shatin, Hong Kong SAR China; 2 Key Laboratory of Combinatorial Biosynthesis and Drug Discovery, Ministry of Education and School of Pharmaceutical Sciences, Wuhan University, 430071 Wuhan, China; 3 School of Life Sciences, The Chinese University of Hong Kong, Shatin, Hong Kong SAR China; 4 J1 Biotech Co., Ltd., 430075 Wuhan, China; 5 Department of Electronic and Computer Engineering, Center of Systems Biology and Human Health, School of Science and Institute for Advanced Study, Hong Kong University of Science and Technology, Clear Water Bay, Kowloon, Hong Kong SAR China; 6 State Key Laboratory of Microbial Metabolism, Joint International Research Laboratory of Metabolic and Developmental Sciences, and School of Life Sciences and Biotechnology, Shanghai Jiao Tong University, Shanghai, 200030 China

**Keywords:** Industrial microbiology, Metabolic engineering, Synthetic biology

## Abstract

Enzymatic reactions in living cells are highly dynamic but simultaneously tightly regulated. Enzyme engineers seek to construct multienzyme complexes to prevent intermediate diffusion, to improve product yield, and to control the flux of metabolites. Here we choose a pair of short peptide tags (RIAD and RIDD) to create scaffold-free enzyme assemblies to achieve these goals. In vitro, assembling enzymes in the menaquinone biosynthetic pathway through RIAD–RIDD interaction yields protein nanoparticles with varying stoichiometries, sizes, geometries, and catalytic efficiency. In *Escherichia coli*, assembling the last enzyme of the upstream mevalonate pathway with the first enzyme of the downstream carotenoid pathway leads to the formation of a pathway node, which increases carotenoid production by 5.7 folds. The same strategy results in a 58% increase in lycopene production in engineered *Saccharomyces cerevisiae*. This work presents a simple strategy to impose metabolic control in biosynthetic microbe factories.

## Abstract (web summary)

Metabolic enzymes often form supramolecular complexes to improve product yield. Here the authors use short peptide tags to create scaffold-free assemblies and synthetic metabolic nodes.

## Introduction

Natural enzymes in their native metabolic pathway are tightly regulated on when and where to exert their activities, and on the concentration of metabolites to produce^1^. This regulation is often lost or at least weakened when enzymes are cloned and expressed in an unfamiliar community of a heterologous host cell or in a test tube. For example, overexpressing metabolic enzymes may lead to imbalanced metabolic flux or the accumulation of toxic intermediates and consequently a stress to the host cell^2–4^. Metabolic engineers seek to increase the flux and reduce the cellular stress by deleting or adding enzymes, adjusting DNA copy numbers, and regulating transcription and translation, as well as including metabolic sensors to install gates and valves on the pathways^5^. Compartmentalization is another way to segregate pathways, avoid metabolic crosstalk, and impose control on the flux. Nature forms membrane-containing or membrane-free organelles, metabolons, multidomain synthases, or multienzyme complexes to achieve a local confinement of the enzymatic activity; examples include *arom* complexes^6^, tryptophan synthase^7^, polyketide synthases^8,9^, and fatty acid synthases^10^ and microcompartments, including carboxysome, encapsulin, lumazine synthase, caveolae, vaults, and others^11^.

**Fig. 1:**
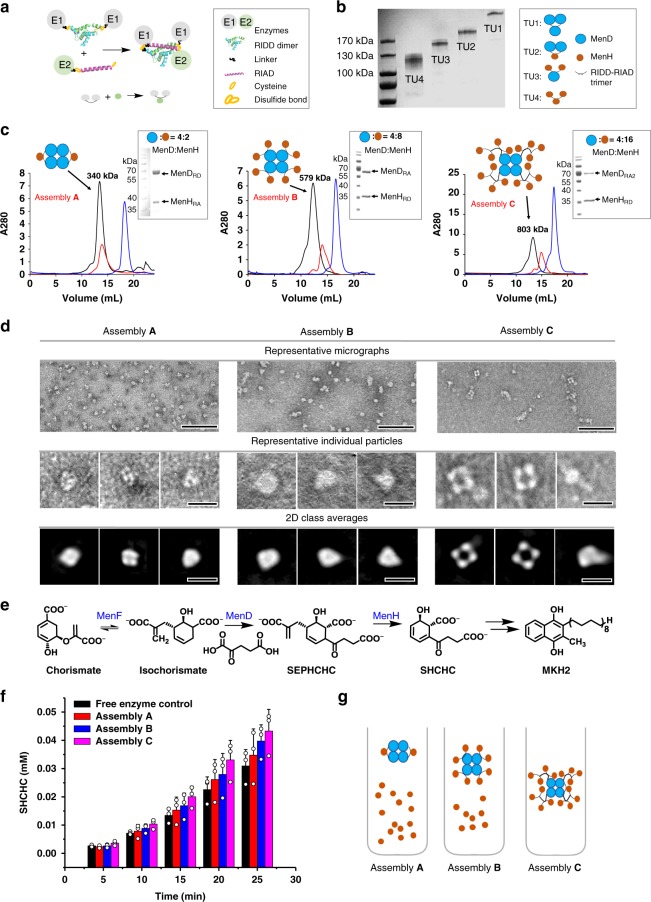
Hierarchical MenD-MenH assemblies mediated by the RIAD–RIDD peptide interaction for biocatalysis. **a** The assembly of tri-enzyme units. E1, E2: enzymes; green and blue structure: RIDD dimer; black line: linker; pink structure: RIAD; one orange circle: cysteine; two orange circles: disulfide bond. **b** Disulfide-stabilized MenD-MenH tri-enzyme units resolved by SDS-PAGE. Blue filled circle: MenD; orange filled circle: MenH; black semilunar line: RIDD-RIAD trimer. **c** Hierarchical enzymes assemblies A, B, and C having different stoichiometries and sizes. Black line: assembly structure; red line: protomers of MenD; blue line: protomers of MenH. **d** Tetrameric structures of the assemblies on TEM. Scale bars: 100 nm (the first row) and 20 nm (the second and third row). **e** MenD and MenH catalyzed conversion of isochorismate to SEPHCHC and then SHCHC. **f** Measurement of the cascade biocatalyst by product generation in three enzyme assembly systems. Red column: Free enzyme control; purple column: Assembly A; blue column: Assembly B; dark blue column: Assembly C. **g** Schematic diagram of Assembly A, B, and C. Error bars indicate the standard deviations of three biological replicates. Source data are provided as a Source Data file

Synthetic multienzyme complexes for local confinement of the enzyme activity have been developed both in vivo^12,13^ and in vitro^14^. For example, enzymes were assembled on a protein scaffold called scaffoldin through dockerin–cohesin interactions as cellulosome-like nano-machineries, and achieved marked increase in catalytic efficiency compared with a mixture of free enzymes^15^. Multidomain protein scaffolds composed of a string of protein-binding domains mediated the assembly of three sequential enzymes in the mevalonate (MVA) biosynthetic pathway through a set of selected protein–peptide interactions. A fine control of metabolic flux and significant improvement in product titer were achieved^12^. However, scaffolded enzyme assemblies are currently known to have different limitations. Enzymes fused in large fusion structures may experience a decrease or complete loss of the activity;^16^ use of DNA or RNA as the scaffolds of multienzyme assemblies is still not generally applicable due to the high cost^17^. The formation of other scaffold filamentous connections may affect cell division^18^. Furthermore, most synthetic multienzyme complexes reported so far are held together by modest interactions^19^. In this report, we have developed a scaffold-free modular enzyme assembly by employing a peptide pair with exceptionally strong affinity but relatively short lengths (Fig. 1a). As a member of the dock-and-lock peptide interacting family^20^, this pair of peptides (RIDD and RIAD) originated from cAMP-dependent protein kinase (PKA) and the A kinase-anchoring proteins (AKAPs), respectively^21,22^. RIDD refers to a docking and dimerization domain of the R subunits of PKAs, the first 44 N-terminal residues. The RIAD peptide is derived from an amphipathic helix of the anchor domain of AKAP that specifically binds to the RIDD dimer^23,24^. The following features make them ideal protein tags for enzyme assembly: (1) the small size (44 and 18 amino acids, respectively), which minimizes the disturbance to the structure, location, and activity of the enzymes when fused as tags, (2) the strong binding affinity (with a *K*_D_ of 1.2 nM between RIDD dimer and RIAD peptide) to ensure the integrity of the enzyme complexes in the cytoplasm, and (3) a 2:1 binding stoichiometry to give branched architectures. RIDD-RIAD-mediated assembly has been validated in the formation of multienzyme complexes in vitro and in the construction of metabolite nodes in the bacterium *Escherichia coli* and the yeast *Saccharomyces cerevisiae* to streamline the flux of carotenoid biosynthesis.

## Results

### Construction of multienzyme complexes in vitro

Modular enzyme assembly was first showcased in vitro using menaquinone biosynthetic enzymes as a model^25^. MenD (2-succinyl-5-enolpyruvyl-6-hydroxy-3-cyclohexadiene-1-carboxylate synthase) from *E. coli* forms a tetramer with each subunit being 63 kDa, and catalyzes the addition of α-ketoglutarate and isochorismate to give 2-succinyl-5-enolpyruvyl-6-hydroxy-3-cyclohexadiene-1-carboxylate (SEPHCHC) with thiamine pyrophosphate as a cofactor. MenH (2-succinyl-6-hydroxy-2,4-cyclohexadiene-1-carboxylate synthase) is a 30 kDa monomer that converts SEPHCHC to 2-succinyl-6-hydroxy-2,4-cyclohexadiene-1-carboxylate (SHCHC) (Supplementary Figs. 1 and 2). No interactions were observed between untagged MenD and MenH when both were mixed in solution. RIAD or RIDD was fused to the C termini of MenD or MenH spaced by a flexible linker (GGGGS)_3_ to give five protomers: MenD_RA_ (MenD-RIAD with one RIAD peptide tag), MenD_RA2_ (MenD-RIAD-RIAD with two sequential RIAD peptide tags), MenD_RD_ (MenD-RIDD with one RIDD peptide tag), MenH_RA_ (MenH-RIAD with one RIAD peptide tag), and MenH_RD_ (MenH-RIDD with one RIDD peptide tag). All five protomers were successfully expressed, purified in soluble forms (Supplementary Fig. 1), and found to retain the same enzymatic activity as the non-tagged enzymes (Supplementary Fig. 3). To facilitate characterization of the hierarchical assembly, the cysteines were introduced to the N termini of RIAD and RIDD to covalently lock the RIAD–RIDD interactions by disulfide bonds and to covalently link tri-enzyme units (TU). TU1 (MenD_3_), TU2 (MenD_2_-MenH), TU3 (MenD-MenH_2_), and TU4 (MenH_3_) were resolved on non-reducing sodium dodecyl sulfate-polyacrylamide gel electrophoresis (SDS-PAGE) with expected molecular weights (Fig. 1b). Under native conditions, the MenD-MenH units assemble to form higher-order architectures mediated by both the RIAD–RIDD interaction and the multimerization state of MenD. Around the tetrameric MenD as the core, we formed 3 assemblies, MenD_4_-MenH_2_ (Assembly A), MenD_4_-MenH_8_ (Assembly B), and MenD_4_-MenH_16_ (Assembly C), each with 4 MenD in the core and 2, 8, or 16 copies of MenH at the periphery, respectively (2, 8, or 16 MenH enzymes with 4 MenD enzymes per particle) (Fig. 1c). The size-exclusion chromatography (SEC) showed expected molecular weights of 340, 579, and 803 kDa. The transmission electron microscopy (TEM) elucidated nano-structures with diameters ranging from 11 to 20 nm (Fig. 1d). The tetrameric cores of MenD were visible in all three assemblies. The enzyme particles of Assembly C showed significantly different morphology than Assemblies A and B, possibly because of the steric hindrance of the high-density MenH enzymes surrounding the MenD core. Two-dimensional single-particle analysis on Assembly B found that the crystal structure of the tetrameric MenD can perfectly fit in the images (Supplementary Fig. 4), but MenH was not visible, possibly due to the flexible movement of MenH incurred by the linker. This result manifests that hierarchical assembly of enzymes using the RIAD–RIDD interacting pair can form structurally independent multienzyme assemblies with tunable compositions.

The catalytic properties of the three MenD-MenH assemblies above were then evaluated. Chorismate and excess amount of isochorismate synthase (MenF) were added in the solution to establish a rapid equilibrium to form isochorismate, the starting material for MenD and MenH (Fig. 1d). To ensure that the reaction solutions contain the same amount of MenD and MenH, we supplemented the assemblies with untagged free MenH enzyme in Assemblies B and C. Because each assembly of A, B, and C contains 4 MenD proteins and 2, 8, and 16 MenH molecules, respectively, 14 copies of free MenH were added in the reaction solution of Assembly A, and 8 copies of free MenH were added the reaction solution of Assembly C so that each reaction solution contained the same amount of MenD and MenH (Fig. 1f, g). The Assembly C system showed the highest production rate of SHCHC (40% more SHCHC than in free enzyme mixture system after 25 min), followed by systems B and A. Therefore, higher degree of enzyme assembly correlates with more efficient intermediate transfer and higher overall catalytic efficiency of the cascade reactions, due to the proximity of the cascade enzymes brought together by enzyme assembly.

### Assembling Idi and CrtE in carotenoid-producing bacteria

**Fig. 2:**
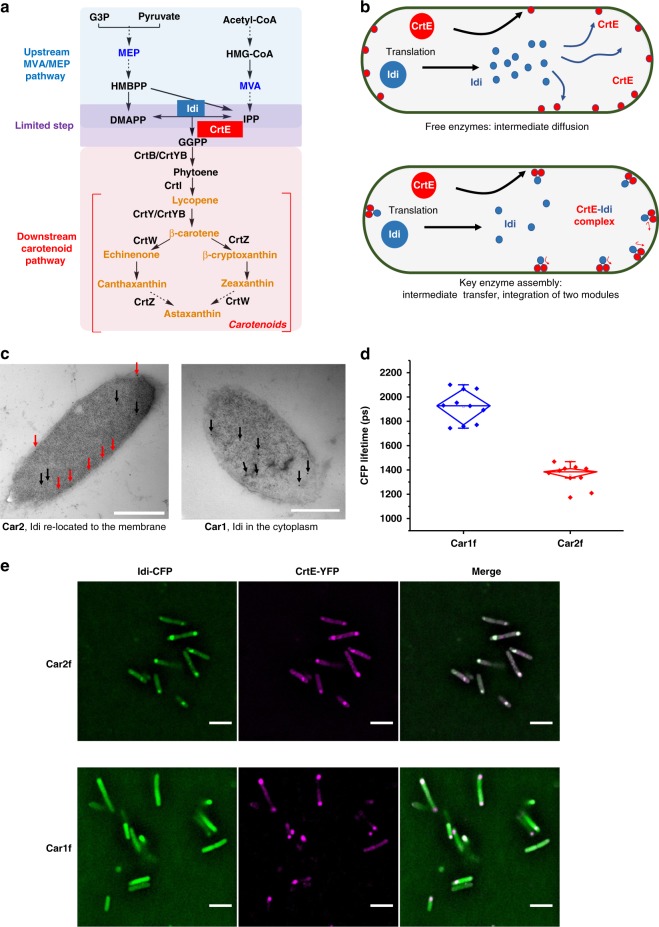
Assembly of the limiting enzymes in carotenoid biosynthesis in the bacterium cells. **a** The Idi and CrtE enzymes represent the limiting step between the two pathways. **b** Physical association of Idi and CrtE changes the transfer of C5 precursors. Red filled circle: CrtE; blue filled circle: Idi; green circle: *E. coli* cells. **c** The RIAD–RIDD peptide interaction induces redistribution of Idi-specific immunogold particles from the cytosol to the plasma membrane. Red arrows: Idi on the plasma membrane; black arrows: Idi in the cytosol. Scale bars: 500 nm. **d** FRET between Idi-CFP and CrtE-YFP measured by fluorescence lifetime imaging microscopy (FLIM). The cells were excited at 810 nm in a two-photon microscope and the fluorescent signals were collected at 481 nm. Error bars indicate the standard deviations of ten replicates. **e** Co-localization of assembled Idi-CFP and CrtE-YFP under the fluorescent microscopy. Scale bars: 2 μm. Source data are provided as a Source Data file

Encouraged by the structural and functional benefits of modular enzyme assembly in vitro, we next applied the assembly strategy inside bacterial cells, specific to carotenoid biosynthesis. Two heterologous pathways were co-transformed in *E. coli* to realize carotenoid biosynthesis: an upstream MVA pathway that converts acetyl-coenzyme A (Ac-CoA) to the five carbon molecules isopentenyl pyrophosphate (IPP) and its allylic isomer dimethylallyl pyrophosphate (DMAPP) in the cytosol, and a downstream carotenoid biosynthetic pathway on plasma membrane responsible for condensing IPP and DMAPP sequentially to carotenoids, such as astaxanthin, zeaxanthin, canthaxanthin, and so on^26^ (Fig. 2a). A critical reaction that links the upstream with the downstream pathways, the reversible interconvertion of IPP and DMAPP, is catalyzed by isopentenyl diphosphate isomerase (Idi). The C5 building blocks IPP and DMAPP are the starting materials for the synthesis of terpenoids in the downstream pathway and also universal precursors for many other metabolites in the cell. Prenyl transferases condenses IPP and DMAPP to yield geranyl pyrophosphate (GPP, C10), then farnesyl pyrophosphate (FPP, C15), and finally geranylgeranyl pyrophosphate (GGPP, C20). These three compounds may then undergo condensation and post modification to give carotenoids catalyzed by other carotenoid enzymes. The supply of IPP and DMAPP poses a bottleneck to the downstream carotenoid biosynthesis, and the excess supply of these C5 building blocks can enhance the production of carotenoids^27^. However, high levels of IPP and DMAPP are known to pose significant cytotoxicity^28,29^. Therefore, heterologous production of carotenoids can decrease cell growth and limit product titers.

We sought to solve this dilemma by enzyme assembly. The last enzyme of MVA pathway Idi has been proven to play an important role in terpene synthesis, such as farnesene (C15)^30^, taxadiene (C20)^31^, lycopene (C40)^32^, and so on. Geranylgeranyl diphosphate synthase (CrtE) as the first enzyme of carotenoid synthetic pathway catalyzes the synthesis of the precursor of carotenoids synthesis—GGPP. We reason that assembly of Idi and CrtE will channel IPP/DMAPP to GGPP synthesis directly to streamline carotenoid biosynthesis. Notably, Idi resides in the cytoplasm, whereas CrtE is membrane bound (this may be part of the reason for inefficient transfer of the IPP and DMAPP to CrtE, as the negatively charged pyrophosphate molecules may be repelled by the plasma membrane, which is also negatively charged). Therefore, we assembled Idi and CrtE as one enzyme complex through the RIAD–RIDD peptide interaction in *E. coli* (Fig. 2b). RIDD and RIAD peptides were fused to the C termini of CrtE and Idi, respectively, thus yielding a bacterium strain Car2, containing CrtE_RD_ (CrtE-RIDD) and Idi_RA_ (Idi-RIAD, also containing a His-tag at the N terminus). CrtE_RD_ and Idi_RA_ spontaneously form an Idi-CrtE enzyme complex (Idi-CrtE_2_) in a 1:2 stoichiometry. Car1 serves as a control; the only difference between Car1 and Car2 is the absence of the RIDD/RIAD peptide tags, but all other enzymes remain identical. Immuno-staining of the His-tagged Idi_RA_ in Car2 cells by an anti-His-tag antibody showed that most of the Idi proteins (59% based on the counts of gold nanoparticles, or 96 out of 162 in 12 images) re-located to the plasma membrane upon assembly (Fig. 2c). By contrast, in the control Car1 stain, the Idi protein was seen mostly in the cytosol (92%, or 58 out of 63 gold nanoparticle counts). Next, we fused Idi_RA_ with cyan fluorescent protein (CFP) to give Idi_RA_-CFP, and CrtE_RD_ with yellow fluorescent protein (YFP) to give CrtE_RD_-YFP in bacterial strain Car2f. A corresponding control strain Car1f was constructed without assembly due to the lack of RIAD or RIDD. Measurement of the fluorescence lifetime of the CFP signal showed significant decrease in Car2f cells than that in Car1f cells (1346.83 vs. 1899.15 ps), indicating a fluorescence resonance energy transfer (FRET) effect due to the proximity of CFP and YFP (the distance between the two chromophores is estimated to be 5.8 nm) (Fig. 2d). Both *E. coli* strains also showed a very different fluorescent signal distribution under the microscope: Idi_RA_-CFP concentrates at the ends of living bacterial cells, and co-localizes with CrtE_RD_-YFP in Car2f strain, whereas in the control stain Car1f, Idi-CFP distributes evenly in the cytosol (Fig. 2e). All these results strongly support that Idi and CrtE have been successfully assembled.

### Increased carotenoid production in shake-flask fermentation

**Fig. 3:**
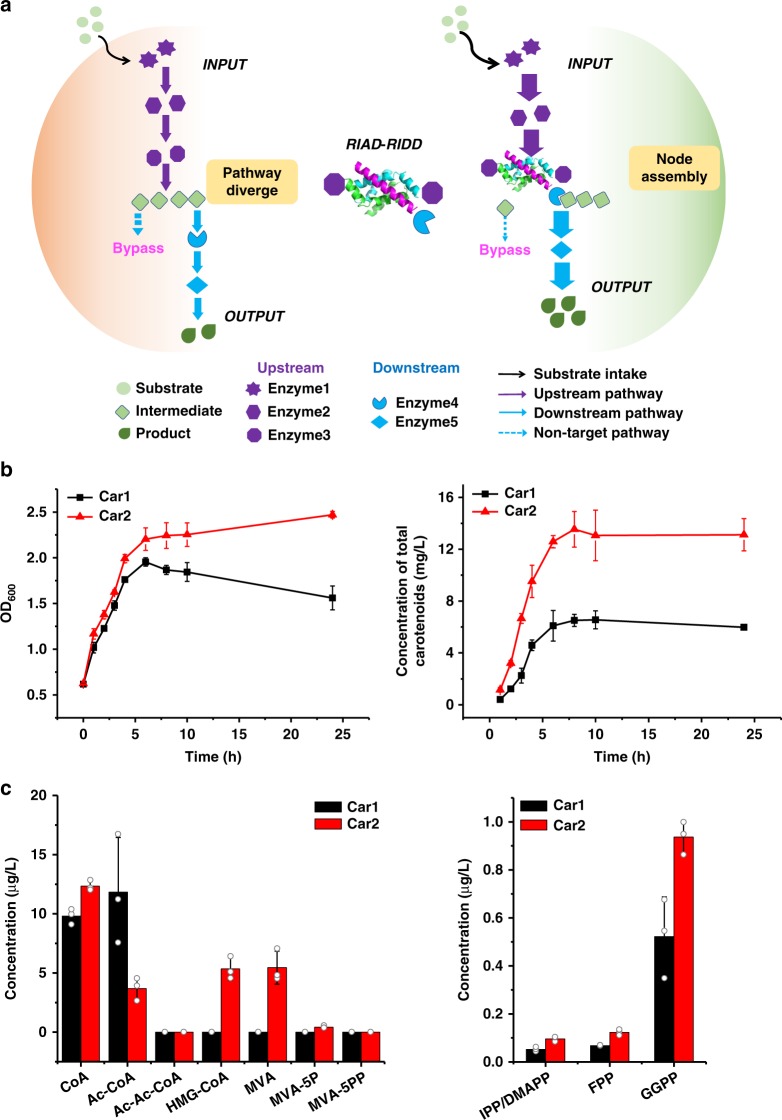
Idi-CrtE assembly increases the metabolic flux of carotenoid biosynthesis in shake-flask fermentation. **a** A scheme showing that the Idi-CrtE assembly installs a metabolic node that guide the flux towards carotenoid synthesis. Green filled circle: substrate; green filled rhombus: intermediate; green drop: product. Seven horns star, hexagon, octagon: enzymes of the upstream. Notched circle, blue filled rhombus: enzymes of the downstream. Black arrow: substrate intake; purple arrow: upstream pathway; blue arrow: downstream pathway; blue dotted arrow: non-target pathway. **b** Enzyme assembly increases carotenoid production and the cell mass. Black line: Car1; red line: Car2. **c** Global changes of the metabolic intermediates. Black column: Car1; red column: Car2. Error bars indicate the standard deviations of three biological replicates. Source data are provided as a Source Data file

We envision that the assembly of Idi and CrtE will install a metabolic node to the carotenoid biosynthesis pathway, streamline carotenoid biosynthesis, and increase the titer (Fig. 3a). We constructed carotenoid-producing strains and measure the production of total carotenoids in shake-flask fermentations. Car2 stain contains an Idi_RA_ and a CrtE_RD_ for assembly of 1:2 Idi-CrtE complex (Supplementary Tables 1 and 2). All other MVA and carotenoid biosynthetic enzymes were the same as in the base strain Car1 but without assembly, which was reported to give an astaxanthin production of 8.64 mg g^−1^ dry cell weight^33^. Impressively, we found that the Car2 strain produced 2.3-fold of the total amount of all the carotenoids, higher than that in Car1 (14.92 vs. 6.44 μg mL^−1^) after 8 h (Fig. 3b). Carotenoid production started to increase after induction, and plateaued after 8 h in both strains. Strikingly, Car2 cells continued to proliferate up to 10 h, whereas Car1 cells ceased to grow after 6 h and started to decline. We ruled out the possibility that C-terminal tagging of Idi or CrtE increases the enzymatic activity as a cause for the higher carotenoid production, by constructing reference strains containing only one of the tags but without the capability to assemble: Car3 has Idi_RA_ but CrtE is untagged; Car4 has CrtE_RD_ but Idi is untagged. Neither strain showed higher cell growth or carotenoid production (Supplementary Fig. 5). The assembly of Idi and CrtE therefore joins the MVA pathway and carotenoid biosynthetic pathway, and more efficiently transfers the C5 precursors to downstream synthesis.

We next compared the levels of the key metabolites in Car2 and Car1 strains by mass spectrometry-based analysis^29^. Key metabolic intermediates are categorized as intermediates of MVA pathway [CoA, Ac-CoA, acetoacetyl-CoA (Ac-Ac-CoA), (*S*)-3-hydroxy-3-methylglutaryl-CoA (HMG-CoA), MVA, mevalonate-5-phosphate (MVA-5P), mevalonate diphosphate (MVA-5PP)] and intermediates of terpene pathway (IPP/DMAPP, FPP, GGPP). Most of the MVA intermediates (CoA, HMG-CoA, MVA, and MVA-5P) increased in Car2 stain, except that Ac-CoA decreased by 70% (the level of Ac-Ac-CoA and MVA-5PP was undetectable) (Fig. 3c). IPP and DMAPP eluted as one peak in liquid chromatography-mass spectrometry (LC-MS), so they were counted together. IPP/DMAPP, FPP, and GGPP in Car2 increased 80% as compared with those in Car1. Idi-CrtE assembly therefore guided the metabolic flux toward the synthesis of carotenoids.

### Increased carotenoid production in fed-batch fermentation

**Fig. 4:**
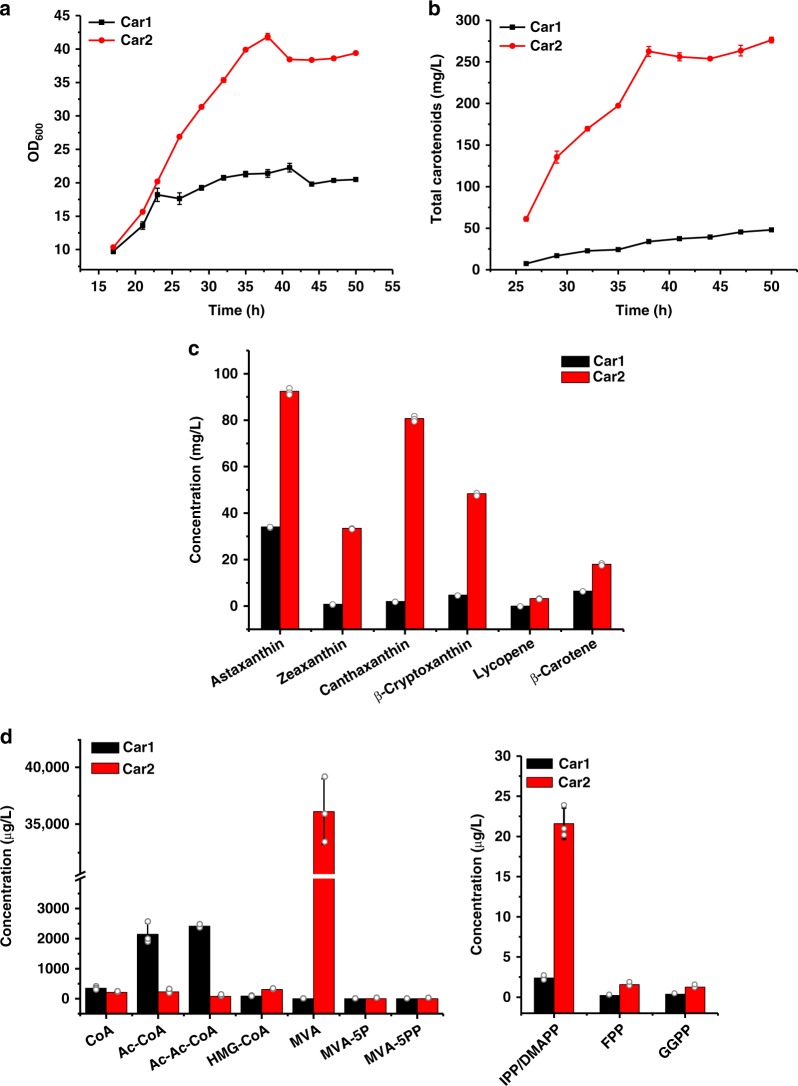
Enzyme assembly gearing metabolic flux towards carotenoid biosynthesis. **a** Comparison of the growth curve of Car2 and Car1 in fed-batch fermentation. **b** Comparison of the yield of overall carotenoids of Car2 and Car1 in fed-batch fermentation. Black line: Car1; red line: Car2. **c** Comparison of the product of main carotenoids. **d** Changes of the metabolic intermediates in responsive to enzyme assembly. Black column: Car1; red column: Car2. Error bars indicate the standard deviations of three replicates. Source data are provided as a Source Data file

We next sought to scale up carotenoid production in 7-L fed-batch fermentation. Although the exact values varied between different fermentations (Supplementary Fig. 6), we observed a robust increase of carotenoid production in Car2. In a representative example, Car2 strain grew up to an OD_600_ of 39.8, whereas the OD_600_ of Car1 strain plateaued at 21 (Fig. 4a). The titer of final product astaxanthin reached 92.4 mg L^−1^ in Car2 strain at 50 h, which was 2.7-fold of that in Car1 (34.1 mg L^−1^). Besides astaxanthin, other carotenoids all showed titer increase ranging from 2.8- to 617-folds, giving a total amount of all carotenoids of 276.3 mg L^−1^ in Car2, 5.7-fold of that in Car1 (48.0 mg L^−1^) (Fig. 4b, c). Measurement of the metabolites showed a similar trend as that in the shake-flask fermentation. Although the content of Ac-CoA and Ac-Ac-CoA decreased, we observed a strikingly high level of MVA, 36.2 mg L^−1^ in Car2, whereas MVA was undetectable in Car1 (Fig. 4d). The IPP/DMAPP level also increased nearly 10-fold in Car2. Therefore, under the conditions of fed-batch fermentation, Idi-CrtE assembly caused a significant, global change of metabolic network in *E. coli*.

### Increased lycopene production in *S. cerevisiea* yeast strain

**Fig. 5:**
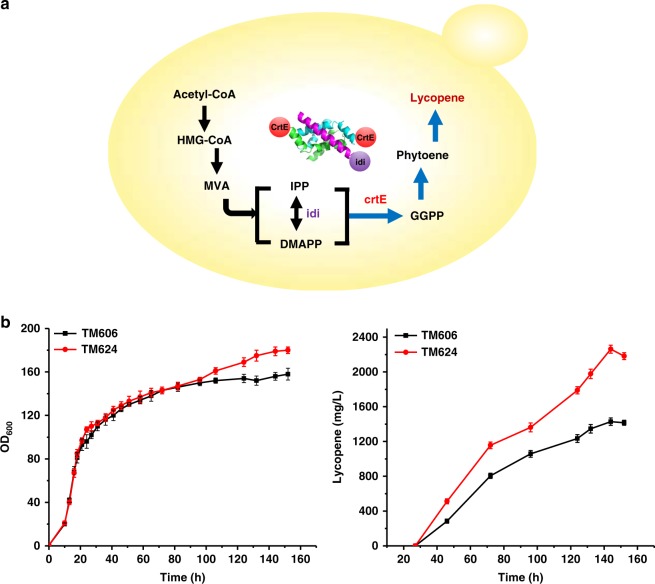
Idi-CrtE assembly increases lycopene production in yeast in fed-batch fermentation. **a** A scheme showing that the Idi-CrtE assembly of lycopene biosynthesis in *S. cerevisiea*. **b** Enzyme assembly increases lycopene production and the cell mass. Black line: TM606; red line: TM624. Error bars indicate the standard deviations of two replicates. Source data are provided as a Source Data file

We next examined whether Idi-CrtE assembly could promote biosynthesis in yeast *S. cerevisiea*. A lycopene-producing yeast strain TM606^34^ was used as a base strain for enzyme assembly. For enzyme assembly in the TM606 strain, the endogenous Idi and one copy of TmCrtE were tagged with RIAD and RIDD, respectively, to give strain TM624. Seven-liter fermentation was carried out to evaluate lycopene production in yeast TM606 and TM624. The assembled strain TM624 gave a yield of 2.3 g L^−1^ lycopene at 144 h fermentation, 58% increase compared to the yield in the unassembled strain TM606. The cell mass of TM624 reached an OD_600_ of 180, also significantly higher than that in TM606 (Fig. 5). We proved that Idi-CrtE assembly also effectively increased lycopene production in yeast and was able to reach a titer of 2.3 g L^−1^, the highest reported lycopene production in *S. cerevisiae* up to date.

## Discussion

Our work highlights a modular assembly of metabolic enzymes to streamline carotenoid biosynthesis in both *E. coli* and *S. cerevisiae*, yielding a titer increase of 5.7-fold titer in *E. coli* and 2.3 g L^−1^ lycopene in *S. cerevisiae*. This work thereby provides a manner that is orthogonal to traditional metabolic engineering: manipulating the physical location of enzymes without changing the activity or abundance of the enzymes holds promise to produce high-yield strains. The relative physical location of Idi and CrtE represents a regulatory factor for heterologous terpene biosynthesis. This discovery will impact the biosynthesis of other terpene products, many of which are highly sought-after, valuable commodities. Although two sequential enzymes can also be physically linked together through direct fusion, we have shown that the enzyme assembly strategy produced complexes with higher catalytic efficiency than enzyme fusions both in the test tube and in *E. coli* cells (Supplementary Figs. 7 and 8).

This work also represents one important step to unveil the mechanism on how natural multienzyme regulates metabolic network through synthetic multienzyme complexes. On the one hand, the globular complexes we assembled in vitro resemble the structures of natural multienzyme complexes such as pyruvate dehydrogenase complexes. Also, atomic resolution structures of the enzyme complexes can help reveal how the peptide interaction drives the formation of the complexes and how substrate channeling could be designed. On the other hand, the crosstalk of metabolic pathways still remains enigmatic. Synthetic metabolic nodes as a way of perturbing the metabolic network likely offer a very useful tool.

## Methods

### Plasmid construction for menaquinone biosynthetic enzymes

Menaquinone biosynthetic genes from *E. coli* were amplified from the stocked plasmids by PCR using primers that introduced restriction enzymes recognition sites *Bam*HI and *Hin*dIII, respectively. The gene of protein interaction interface (RA, short for RIAD; and RD, short for RIDD) with a DNA sequence encoding a N-terminal (GGGGS)_3_ linker sequence was synthesized by Beijing Genomics Institute (5′ *Hin*dIII site and 3′ *Xho*I site were incorporated into the synthesized genes, see details in Supplementary Information). The double enzyme-digested gene fragments were inserted into modified pET32a (pETM, a DNA fragment coding a start codon, a hexahistidine tag, and a thrombin site replaces the sequence from the start codon of the thioredoxin gene to the last nucleotide of the *Eco*RV restriction site of the vector) vector sequentially. One more copy of RA and linker sequence was generated by overlapping PCR. The resultant recombinant vectors named as pETM-MenD_RA_, pETM-MenD_RA2_, pETM-MenD_RD_, pETM-MenH_RA_, and pET-MenH_RD_ were transformed into BL21(DE3) for overexpression and purification of recombinant menaquinone enzymes, respectively. The detailed information of the recombinant plasmids was listed in Supplementary Tables 1–4.

### Formation and purification of multienzyme complexes

A colony picked from agar plate was inoculated into 5 mL Lysogeny broth (LB) supplemented with 100 μg mL^−1^ ampicillin and incubated overnight. Four-milliliter aliquots of cell culture overnight were then inoculated into 400 mL LB containing 100 μg mL^−1^ ampicillin. Cells were incubated at 37 °C and spun at 220 r.p.m. When the OD_600_ of the culture was ~0.6, 0.3 mM final concentration of isopropyl β-d-1-thiogalactopyranoside (IPTG) was added into the cell culture to induce the overexpression of recombinant enzymes and grown overnight at 16 °C and spun at 180 .r.p.m. Then, the cultures were harvested by using centrifugation at 4500 × *g* for 10 min at 4 °C, and then re-suspended in 50 mM Tris (pH 8.0) buffer containing 150 mM NaCl, 10 mM imidazole, and 4 mM 2-mercaptoethanol, and finally lysed with a sonicator. The fusion enzymes in the supernatant were further purified to homogeneity by using HisTrap HP (GE Healthcare Life Sciences). The purified proteins were concentrated to about 5 mg mL^−1^ by using Amicon Ultra-15 Centrifugal Filter Units (Merck). Then, the proteins were stocked under −80 °C with Tris buffer (pH 7.8) containing 50 mM NaCl and 10% glycerol (v/v) for following construction of multienzyme complexes. Uncropped scans of the blots have been given in the Source Data file.

To construct multienzyme complexes in vitro, 1.5 mg of enzyme fused with RIAD and two-fold molar excess of enzyme fused with RIDD were mixed together in Tris buffer (pH 7.8) containing 1 mM EDTA and 0.02% Tween-20. Without incubation, the mixture was loaded onto Superdex 200 10/300 GL for purification. SEC was performed on ÄKTA prime plus with a flow rate of 0.4 mL min^−1^ in Tris buffer (pH 7.8) containing 50 mM NaCl. The purified multienzyme complexes were concentrated by using Amicon Ultra-15 Centrifugal Filter Units and stored with 10% glycerol (v/v) under −80 °C.

### Establishment of calibration curve of standard proteins

Individual proteins standard purchased from Sigma was subjected onto Superdex 200 10/300 GL on ÄKTA prime plus to determine the respective elution volume (*V*_e_) with a flow rate of 0.4 mL min^−1^ in Tris buffer (pH 7.8) containing 50 mM NaCl. The retention volume of blue Dextran (2000 kDa) was the indicator of the void volume (*V*_o_) of the column. Plot log molecular weight (log MW) vs. *V*_e_/*V*_o_ for each respective protein standard and then the molecular weight of unknown protein and protein complexes were determined from the standard curves.

### Plasmid construction for carotenoid biosynthesis

The parental plasmids pMH1 and pFZ81 containing the genes involved in the MVA pathway and pFZ153 consisting of genes for carotenoids biosynthesis were used. By using overlapping PCR, genes of protein-binding domains were attached to the C terminals of selected enzyme genes via a DNA sequence, which encodes for a (GGGGS)_3_ linker, respectively. Using Gibson assembly^35^, the resultant fusion genes were then inserted into the parental plasmids to replace the genes that encodes for freely floating wild-type enzymes in parental plasmids (see details in Supplementary Table 1).

### ImmunoTEM

*Escherichia coli* cells were inoculated in LB medium and grown until the OD_600_ reached 0.4–0.6. Cells in the cultures were concentrated by filtering through cellulose membranes with 0.2 μm pores and frozen with an HPM100 high-pressure freezer (Leica Microsystems). Resin embedding, ultramicrotomy, and immunogold labeling were performed as described before^36^. Briefly, the frozen specimens were freeze substituted in anhydrous acetone containing 0.25% glutaraldehyde and 0.1% uranyl acetate at −80 °C for 24 h and slowly warmed up to −45 °C. After rinsing with precooled acetone, the cells were embedded in Lowicryl HM20 resin at −45 °C and the resin was polymerized by ultraviolet illumination. Ultrathin (100 nm) sections from the samples were collected on nickel slot grids coated with formvar. The sections were probed with a mouse anti-His monoclonal antibody (Vendor) and gold partible (15 nm) conjugated secondary antibody (Ted Pella).

### Lifetime-based FRET

FRET is used as a molecular ruler due to its sensitivity in the range of 2–10 nm. In contrast to standard FRET measuring changes of the fluorescence intensity, lifetime-based FRET enables quantitative analysis by using the fluorescence lifetime of the donor molecule as a probe that is in a broad range, concentration independent. This is crucial since in biological systems-like cells the fluorophore concentration often cannot be accurately determined and compared among different cells. The fluorescence lifetime of the donor is effectively decreased (quenched) when it undergoes FRET with an acceptor molecule. Thus, comparing the donor lifetimes in the absence and presence of the acceptor provides information about the FRET efficiency.

The fluorescence lifetime of CFP (donor) was measured by a home-built two-photon fluorescence lifetime imaging microscope. The CFP fluorescence was excited by a 810-nm femtosecond laser and its lifetime was recorded by a time-correlated single photon counting module. The measured CFP lifetimes in Car2f and Car1f cells were *τ*_DA_ = 1346.83 ps and *τ*_D_ = 1899.15 ps, respectively. The FRET efficiency *E* can then be easily calculated according to the following equation: *E* = 1 − *τ*_DA_/*τ*_D_, *E* = 29%. The CFP-YFP distance was 5.8 nm by using the following equation (*R*_0_ is 5 nm for CFP/YFP pairs^37^):

1 $$R = R_0\root {6} \of {{(1 - E)/E}}.$$

### Fermentation of carotenoid-producing strains

To create the seed culture, several single colonies of strains were grown in 6 mL LB (with appropriate antibiotics 50 mg L^−1^ kanamycin, 100 mg L^−1^ ampicillin, 34 mg L^−1^ chloramphenicol) medium overnight at 30 °C in a rotary shaker at 220 r.p.m.

For shake-flask fermentation, subcultured 1% of the culture in 200 mL LB (with appropriate antibiotics) at 30 °C and spun at 220 r.p.m. When the OD_600_ reached 0.6–0.8, IPTG was added to a final concentration of 0.1 mM for induction. One milliliter of culture was harvested and stored at −40 °C for analysis of carotenoid accumulation. Four hundred OD_600_ cells of each engineered strain were harvested at 8 h after induction, immediately quenched and extracted by methanol: 0.5% NaCl = 2:1 (v/v), suspended the pellets, and centrifuged at 5000 × *g* for 3 min at 4 °C. The pellet was collected and stored at −40 °C for intermediate accumulation of MVA pathway analysis.

For fed-batch fermentation, subcultured 1% of the seed culture in 2 × 150 mL LB (with appropriate antibiotics) at 30 °C and spun at 220 r.p.m. until OD_600_ reached 2. Then, 300 mL of the seed culture was inoculated into 2.5 L M9 medium (10 g L^−1^ (NH_4_)_2_SO_4_, 8.5 g L^−1^ KH_2_PO_4_, 1 g L^−^^1^ MgSO_4_·7H_2_O, 0.5 g L^−^^1^ sodium citrate, 30 g L^−1^ glycerol, 0.07 g L^−1^ CaCl_2_·2H_2_O, 10 mL L^−1^ thiamine solution, 4 mL L^−1^ vitamins, 4 mL L^−1^ metals solutions, and appropriate antibiotics)^38^ in 7-L fermenter for fed-batch fermentation. Fed-batch fermentation was maintained at 30 °C and pH was maintained at 7.0 using 9.9 N NH_4_OH (NH_4_OH:H_2_O = 2:1, v/v). The initial agitation was set to 200 r.p.m. and the foam was controlled by adding antifoam THIX-298. When OD_600_ reached ∼10, the culture was fed a sterile glycerol (feed solution: 500 g L^−1^ glycerol, 2 g L^−1^ MgSO_4_·7H_2_O) to meet the cell growth. Dissolved oxygen tension was maintained above 20% by adjusting the agitation (200–750 r.p.m.) and gas flow (0.5–2 vvm (volume of air under standard conditions per volume of liquid per minute)) before induction. IPTG was added to a final concentration of 0.25 mM at cell density (OD_600_) reached ∼20. Ten milliliter-fermentation broth samples were collected by periodic withdrawal and stored at −40 °C for carotenoids analysis. Four hundred OD_600_ cells were harvested when the cells accumulated the highest production of astaxanthin, and immediately quenched and extracted as above for intermediate accumulation of MVA pathway analysis.

### Analysis assay of carotenoids

Cells in culture were harvested at 5000 × *g* for 2 min. The harvested cells were washed with 1 mL water followed by centrifugation at 5000 × *g* for 2 min. The supernatant was discarded. Before adding extraction regent, the pellets were dispersed by vortexing for 5 min. Then, the pellets were extracted with acetone (with 1% butylated hydroxytoluene) by vortexing the solution for 5 min. The solution was centrifuged at 5000 × *g* for 2 min. The supernatant was transferred to a new tube, and the pellets were extracted as above until colorless. The extraction was pooled together and centrifuged at 12,000 × *g* for 10 min. The supernatant was transferred to sample vials for high-performance liquid chromatography (HPLC) measurements. The processes above were carried out at 4 °C.

HPLC (UltraMate 3000, Thermo Scientific) equipped with C18 column (4.6 × 150 mm^2^, 5 μm, Agilent Technologies, Inc.) was used to detect 474, 468, and 450 nm signals at 30 °C. Samples were eluted with the following gradient program with solvent A: 90% aqueous acetonitrile (HPLC grade); solvent B: methyl alcohol-isopropyl alcohol (3:2, v/v, HPLC grade) as the mobile phase at a flow rate of 1.0 mL min^−1^: 0–90% solvent B (0–15 min), maintained 90% solvent B (15–30 min), 90–0% solvent B (30–35 min)^39^. The titers of carotenoids were calculated using a standard curve with an appropriate dilution factor and averaged for three replicate analyses.

### Analysis assay of intermediates in MVA pathway

The quenched pellets were extracted with 1.5 mL of ethanol:acetonitrile:H_2_O = 2:2:1 (v/v) by vortexing for 5 min. The samples were quick frozen with liquid nitrogen and then melted on ice for 4 min and vortexed for 30 s. This freeze–thaw cycle was repeated three times, followed by centrifugation at 12,000 × *g* for 10 min at 4 °C. The supernatant (1.1 mL) was transferred into a clean tube and freeze dried. The residues were suspended with acetonitrile:H_2_O = 50:50 (v/v), and centrifuged at 12,000 × *g* for 10 min at 4 °C. The supernatant was transferred to sample vials for LC-MS measurements.

In MVA pathway, there are 17 intermediates; we constructed three methods with high-resolution LC-MS to analyze them more accurately. The concentrations of intermediates were calculated using a standard curve with appropriate dilution factor and averaged for three replicate analyses.

For separating of IPP/DMAPP, MVA, MVA-5P, and MVA-5PP, Luna 3 μm NH_2_ column (150 × 2 mm^2^, 3 μm) was used. Samples were eluted at 35 °C with the following gradient program with solvent A: 5% aqueous acetonitrile with 10 mM ammonium acetate (pH = 9.5); solvent B: acetonitrile as the mobile phase at a flow rate of 0.2 mL min^−1^: 80% solvent B (0–1 min), 80–5% solvent B (1–8 min), 5% solvent B (8–15 min), 5–80% solvent B (15–15.1 min), 80% solvent B (15.1–21 min). MS was set as electrospray ionization (ESI)-negative full MS/dd-MS2 mode. Full MS was set as follows: 100–1500*M*/*Z* and resolution: 70,000. DD-MS2 was set as follows: resolution, 17,500; isolation window, 1.4*M*/*Z*; stepped (N) CE, 30. Targeted compounds were included in the inclusion list. Other parameters were as follows: spray voltage, 3.1 kV; capillary temperature, 320; aux gas heat temp, 300; sheath gas, 35; aux gas, 5; tray temperature, 8.

For separating of FPP and GGPP, ACE Ultra Core 2.5 Super C18 column (100 × 2.1 mm^2^, 2.5 μm) was used. Samples were eluted at 35 °C with the following gradient program with solvent A: 10 mM ammonium acetate (pH = 9.5) and solvent B: acetonitrile as the mobile phase at a flow rate of 0.2 mL min^−1^: 5–90% solvent B (0–10 min), 90% solvent B (10–11 min), 90–5% solvent B (11–11.1 min), 5% solvent B (11.1–15 min). MS condition was the same as IPP detection.

For separating of CoA, Ac-CoA, Ac-Ac-CoA, HMG-CoA, Thermo Fisher hypercard column (100 × 2.1 mm^2^, 3 μm) was used. Samples were eluted at 35 °C with the following gradient program with solvent A: 10 mM ammonium acetate (pH = 9.5) and solvent B: acetonitrile as the mobile phase at a flow rate of 0.2 mL min^−1^: 5% solvent B (0–1 min), 5–50% solvent B (1–10 min), 50–90% solvent B (10–12 min), 90–5% solvent B (12–12.1 min), 5% solvent B (12.1–16 min). MS was set as ESI-positive full MS/dd-MS2 mode. Spray voltage was −3.1 kV. Other set and parameters were the same as IPP detection.

### Engineering *S. cerevisiea* for lycopene production

The strains constructed in *S. cerevisiea* are listed in Table S2. The primers used for fragments, and the fragments used for strain construction are listed in Tables S3 and S4, respectively. All fragments obtained by polymerase chain reaction were gel purified using a kit (Axygen; Corning Life Science, Corning, NY, USA) before cloning. Yeast cells were transformed using lithium acetate and PEG4000^40^ for assembly cloning and gene deletion. Fragment assembly was performed using the Gibson method^35^ or yeast assembly^41^.

*Saccharomyces cerevisiae* CEN.PK2-1D was used as the background strain for all constructs, and *E. coli* DH10B was used to propagate the recombinant plasmids. Engineered yeast strains were selected on synthetic complete medium [0.67% yeast nitrogen base with (NH_4_)_2_SO_4_, 2% glucose, and appropriate amino acids] under auxotroph-screening conditions (uracil 20 mg L^−1^, histidine 20 mg L^−1^, tryptophan 20 mg L^−1^, and leucine 100 mg L^−1^) or yeast extract–peptone–dextrose medium (2% tryptone, 1% yeast extract, and 2% glucose) with antibiotic screening (G418 200 mg L^−1^ and hygromycin 200 mg L^−1^). All media were autoclaved at 115 °C for 30 min before use. Lycopene standard was purchased from Sigma-Aldrich (St. Louis, MO, USA).

### Lycopene extraction and titration

For lycopene extraction, cells in 0.5 mL of culture were harvested by centrifugation at 5000 × *g* for 5 min, and the supernatants were removed completely. Glass beads (0.2 g; 0.5 mm in diameter) were added, followed by dispersal of the pellets by vortexing. The pellets were then extracted with acetone (using 1% butylated hydroxytoluene) by vortexing until residues were colorless, followed by centrifugation of solution at 13,000 × *g* for 10 min. The supernatant was transferred to sample vials for HPLC measurements, where the lycopene titer was calculated using a standard curve with an appropriate dilution factor and averaged over three replicates. HPLC analysis was carried out in the analysis of carotenoids mentioned above.

### Fermentation of the lycopene-producing yeast strain

Lymphocyte growth medium (LGM) medium was used for fed-batch fermentation. The medium contained (per liter): glucose 20 g, (NH_4_)_2_SO_4_ 15 g, KH_2_PO_4_ 8 g, MgSO_4_·7H_2_O 6.15 g, 50 mM succinate, vitamin solution 12 mL, trace metals solution 10 mL, and NH_3_·H_2_O was used to adjust pH to 5.0. Vitamin solution contained (per liter): biotin 0.05 g, calcium pantothenate 1 g, nicotinic acid 1 g, myo-inositol 25 g, thiamine·HCl 1 g, pyridoxal·HCl 1 g, and *p*-aminobenzoic acid 0.2 g. Trace metals solution contained per liter: EDTA 15 g, ZnSO_4_·7H_2_O 5.75 g, MnCl_2_·4H_2_O 0.32 g, anhydrous CuSO_4_ 0.5 g, CoCl_2_·6H_2_O 0.47 g, Na_2_MoO_4_·2H_2_O 0.48 g, CaCl_2_·2H_2_O 2.9 g, and FeSO_4_·7H_2_O 2.8 g.

To create the seed culture, several single colonies of the lycopene-producing strain were grown in 5 mL LGM medium overnight at 30 °C in a rotary shaker at 220 r.p.m., after which 1% of the seed culture was transferred to a 500-mL flask containing 200 mL LGM medium and cultured at 30 °C with shaking at 220 r.p.m. for 16–18 h. Ten percent of the seed culture was then inoculated into 3-L LGM medium in a 7-L fermenter for fed-batch fermentation at 30 °C, with the pH maintained at 5.0 using NH_3_·H_2_O. Fermentation was performed at an agitation speed between 300 and 600 r.p.m. and an airflow rate ranging from 1.5 to 2 vvm.

We employed a two-stage fed-batch strategy. In the first stage, a feeding solution containing 500 g L^−1^ glucose, 9 g L^−1^ KH_2_PO_4_, 5.12 g L^−1^ MgSO_4_·7H_2_O, 3.5 g L^−1^ K_2_SO_4_, 0.28 g L^−1^ Na_2_SO_4_, 12 mL L^−1^ vitamin solution, and 10 mL L^−1^ trace metals solution was used to achieve rapid cell growth. Following decreases in glucose concentration to ~1 g L^−1^ in the batch culture, feeding was started in order to maintain the residual glucose concentration at between 1 and 2 g L^−1^. When the cell mass began to slowly increase (to stationary phase), the first stage was stopped, and the inducer galactose was added to a final concentration of 10 g L^−1^. In the second stage, the feeding solution ethanol was used to produce lycopene, with the feeding rate adjusted in order to maintain the ethanol concentration at ~5 g L^−1^. When the color of the culture changed, we detected the product in order to assess lycopene accumulation. When the lycopene concentration stopped increasing, fermentation was stopped.

### Reporting summary

Further information on research design is available in the Nature Research Reporting Summary linked to this article.

## Supplementary information

Reporting Summary

Supplementary information

Source Data

## Data Availability

The plasmids generated during the current study and the data that support the findings of this study are available from the corresponding author upon reasonable request. The source data underlying Figs. 1b–d, 1 f, 2c–e, 3b, c, 4a–c, and 5b and Supplementary Figs. 1–5, 6a, b, 7, and 8b are provided as a Source Data file.
