# Supplementary material for: Modular enzyme assembly for enhanced cascade biocatalysis and metabolic flux
Source: Nat Commun. 2019 Sep 18;10:4248. doi: 10.1038/s41467-019-12247-w (PMC6751169; doi:10.1038/s41467-019-12247-w)
Supplement: Supplementary file 2 — Supplementary information [file 41467_2019_12247_MOESM2_ESM.pdf]

Modular enzyme assembly for enhanced cascade biocatalysis and metabolic flux

Kang and Ma et al.

Contents

**Items**	**Page No.**
**Supplementary Table 1. Plasmids used in this study**	**S3**
**Supplementary Table 2. Strains used in this study**	**S5**
**Supplementary Table 3. Strains and plasmids construction**	**S6**
**Supplementary Table 4. Primers used in this study**	**S11**
**Supplementary Figure 1.** Purified $MenH_{RD}$ (lane 1), $MenH_{RA}$ (lane 2), $MenD_{RA}$ (lane3), $MenD_{RA2}$ (lane 4) and $MenD_{RD}$ (lane 5) analyzed by SDS-PAGE.	**S14**
**Supplementary Figure 2.** Purified MenD elutes as a tetramer, and MenH as a monomer on Superdex 200 10/300 GL in SEC.	**S15**
**Supplementary Figure 3.** Peptide tagging did not change the activity of MenD and MenH.	**S16**
**Supplementary Figure 4.** TEM images of the Assembly B.	**S17**
**Supplementary Figure 5.** Strains Car3 and Car4 have identical carotenoids production rates and growth rates as the untagged control Car1 in shake-flask fermentation.	**S18**
**Supplementary Figure 6.** Independent fed-batch fermentations showing the same trend of carotenoid production.	**S19**
**Supplementary Figure 7.** Comparison of enzyme activity of the MenD-MenH fusion enzyme and free MenD and MenH in 1:1 ratio, showing the enzyme fusion has significantly reduced activity.	**S20**
**Supplementary Figure 8.** Comparison of the assembly strains and fusion strains, showing that strains with enzyme assembly have higher product titer during shake-flask fermentation.	**S21**
**Supplementary References**	**S22**

Supplementary Table 1. Plasmids used in this study

Plasmids	Backbone	Description	Source
pETM-MenD$_{RA}$	pET32a(+)	N-terminal His-tagged MenD, (GGGGS)$_3$ linker and RIAD, Amp$^+$	this study
pETM-MenD$_{RD}$	pET32a(+)	N-terminal His-tagged MenD, (GGGGS)$_3$ linker and RIDD, Amp$^+$	this study
pETM-MenH$_{RA}$	pET32a(+)	N-terminal His-tagged MenH, (GGGGS)$_3$ linker and RIAD, Amp$^+$	this study
pETM-MenH$_{RD}$	pET32a(+)	N-terminal His-tagged MenH, (GGGGS)$_3$ linker and RIDD, Amp$^+$	this study
pETM-MenD$_{RA2}$	pET32a(+)	N-terminal His-tagged MenD, (GGGGS)$_3$ linker, RIAD, (GGGGS)$_3$ linker and RIAD, Amp$^+$	this study
pETM-MenD-MenH	pET21a(+)	N-terminal His-tagged MenD, (GGGGS)$_3$ linker, MenH, Amp+	this study
pMH1	pBBR1MCS-1	AtoB, ERG13 and N-terminal His-tagged tHMG1, Cam$^+$	reference[1]
pFZ81	pBBR1MCS-2	ERG12, ERG8, MVD1 and N-terminal His-tagged Idi, Kan$^+$	reference[1]
pFZ153	pETDuet-1	CrtE, CrtI, CrtB, Idi, CrtY, CrtZ, CrtW, Amp$^+$	reference[2]
pFZ82	pBBR1MCS-2	ERG12, ERG8, MVD1 and N-terminal His-tagged Idi$_{RA}$, Kan$^+$	this study
pFZ1532	pETDuet-1	CrtE$_{RD}$, CrtI, CrtB, Idi, CrtY, CrtZ, CrtW, Amp$^+$	this study
pFZ81f	pBBR1MCS-2	ERG12, ERG8, MVD1 and N-terminal His-tagged Idi-CFP, Kan$^+$	this study

pFZ82f	pBBR1MCS-2	ERG12, ERG8, MVD1 and N-terminal His-tagged Idi_{RA}-CFP, Kan^+	this study
pFZ1532f	pETDuet-1	$CrtE_{RD}$-YFP, CrtI, CrtB, Idi, CrtY, CrtZ, CrtW, Amp^+	this study
pFZ1530	pETDuet-1	$CrtE_{RD}$, CrtI, CrtB, CrtY, CrtZ, CrtW, Amp^+	this study
pFZ15301	pETDuet-1	CrtE-Idi, CrtI, CrtB, CrtY, CrtZ, CrtW, Amp^+	this study
pFZ15302	pETDuet-1	Idi-CrtE, CrtI, CrtB, CrtY, CrtZ, CrtW, Amp^+	this study
pFZ15322	pETDuet-1	$CrtE_{RD}$, CrtI, CrtB, $Idi_{RA,}$ CrtY, CrtZ, CrtW, Amp^+	this study

Supplementary Table 2. Strains used in this study

Strains	Description	Source
Car1	*E. coli* MG1655 (DE3) with pMH1, pFZ81 and pFZ153	this study
Car2	*E. coli* MG1655 (DE3) with pMH1, pFZ82 and pFZ1532	this study
Car3	*E. coli* MG1655 (DE3) with pMH1, pFZ82 and pFZ153	this study
Car4	*E. coli* MG1655 (DE3) with pMH1, pFZ81 and pFZ1532	this study
Car1f	*E. coli* MG1655 (DE3) with pMH1, pFZ81f and pFZ1532f	this study
Car2f	*E. coli* MG1655 (DE3) with pMH1, pFZ82f and pFZ1532f	this study
Car5	*E. coli* BL21 (DE3) with pMH1, pFZ81 and pFZ15322	this study
Car6	*E. coli* BL21 (DE3) with pMH1, pFZ81 and pFZ15301	this study
Car7	*E. coli* BL21 (DE3) with pMH1, pFZ81 and pFZ15302	this study
TM606	CEN.PK2-1D, *gal1Δ, gal7Δ, gal10Δ:: TRP1*_P_{GAL10}-*tHMG1*<br>*leu2Δ:: LEU2*_P_{GAL1}-Tm*CrtE*<br>*ura3Δ:: HIS3*_ P_{GAL1}-Pa*CrtB,* P_{GAL10}-Bt*CrtI*<br>*YPRCdelta15Δ:: KanMX*_P_{GAL7}-Bt*CrtI,* P_{GAL10}-Tm*CrtE*,<br>*XI-3Δ::* P_{GAL1}-*POS5*,<br>*X-3Δ::* P_{GAL1}-*ADH2,* P_{GAL10}-Se*ACS,* P_{GAL7}-*ALD6,*<br>*ypl062wΔ:: HphMX,*<br>*exg1Δ:: KanMX*	reference[3]
TM624	TM606, Idi::*Idi*-RIAD, Tm*CrtE*:: Tm*CrtE*-RIDD	this study

Supplementary Table 3. Strains and plasmids construction

Plasmids	Fragments	Template	Primers
pETM-MenD$_{RA}$	MenD	*E. coli*	MenD-F<br>MenD-R
pETM-MenH$_{RA}$	MenH	*E. coli*	MenH-F<br>MenH-R
pETM-MenD$_{RA2}$	Fragment 1*	pETM-MenD$_{RA}$	MenD$_{RA2}$-1-F<br>MenD$_{RA2}$-1-R
	Fragment 2	pETM-MenD$_{RA}$	MenD$_{RA2}$-2-F<br>MenD$_{RA2}$-2-R
	Fragment 3	pETM-MenD$_{RA}$	MenD$_{RA2}$-3-F<br>MenD$_{RA2}$-31-R
	MenD$_{RA}$	Fragment 3	MenD$_{RA2}$-3-F<br>MenD$_{RA2}$-32-R
	MenD$_{RA2}$	MenD$_{RA}$	MenD$_{RA2}$-3-F<br>MenD$_{RA2}$-33-R
pFZ82	Idi	pFZ81	82-1-F<br>82-1-R
	RIAD	Synthesis[†]	82-2-F<br>82-2-R
	Idi-RIAD	Idi+RIAD	82-1-F<br>82-2-R
	Fragment 1	pFZ81	82-3-F<br>82-3-R
	Fragment 2	pFZ81	82-4-F<br>82-4-R
pFZ1532	CrtE	pFZ153	1532-1-F<br>1532-1-R
	RIDD	Synthesis[†]	1532-2-F<br>1532-2-R
	CrtE-RIDD	CrtE+RIDD	1532-1-F<br>1532-2-R
	Fragment 1	pFZ153	1532-3-F<br>1532-3-R
	Fragment 2	pFZ153	1532-4-F<br>1532-4-R
pFZ81f	CFP	Synthesis[†]	81f-1-F<br>81f-1-R
	Linker-CFP	CFP	81f-11-F<br>81f-1-R
	Fragment 1	pFZ81	81f-2-F<br>81f-2-R
	Fragment 2	pFZ81	81f-3-F<br>81f-3-R
pFZ82f	CFP	pFZ81f	81f-1-F

			82f-1-R
	RIAD-Fragment 1	pFZ82	82f-2-F
			82f-2-R
	Fragment 2	pFZ81f	82f-3-F
			82f-3-R
pFZ1532f	YFP	Synthesis†	1532f-1-F
			1532f-1-R
	RIDD	Synthesis	1532f-2-F
			1532f-2-R
	Fragment 1	pFZ1532	1532f-3-F
			1532f-3-R
	Fragment 2	pFZ1532	1532f-4-F
			1532f-4-R
pFZ1530	F-1530	pFZ153	1530-F
			1530-R
pFZ15322	Idi_{RA}	pFZ82	15322-1-F
			15322-1-R
	F-CrtYZW	pFZ153	15322-2-F
			15322-2-R
pFZ15301	F-CrtE	pFZ153	15301-1-F
			15301-1-R
	Idi	pFZ153	15301-2-F
			15301-2-R
	F-CrtI	pFZ153	15301-3-F
			15301-3-R
pFZ15302	Idi	pFZ81	15302-1-F
			15302-1-R
	F-CrtEIB	pFZ153	15302-2-F
			15302-2-R

Strains

TM624	CrtE left	TM606	crtE_left-F
			RI-crtE R
	RIDD	pFZ1532	crtE-RI F
			GPM RI R
	tGPM	TM606	RI-GPM F
			crtE_RI-terminator-R
	HghMX	pUG75	crtE_hygR-F
			crtE_hygR-R
	CrtE right	TM606	crtE_right-F
			crtE_right-R
	Idi left	TM606	idi left F
			RIAD idi R
	RIAD	pFZ82	idi RIAD F

		Ter RIAD R
Ter	TM606	RIAD Ter F
		G418 Ter R
KanMX	pUG6	TER G418 F
		MRP G418 R
Idi right	TM606	G418 MRP F
		MRP R

*Underline was the fragments used in Gibson assembly.

†Sequence of RIAD:
GGTGGTGGTGGTTCAGGTGGTGGTGGTTCAGGTGGTGGTGGTTGTGGTCTGGAAC
AGTATGCAAATCAGCTGGCAGATCAGATTATCAAAGAAGCAACCGAAGGTTGC
Sequence of RIDD:
GGTGGTGGTGGTTCAGGTGGTGGTGGTTCAGGTGGTGGTGGTTGTGGTAGCCTGC
GTGAATGTGAACTGTATGTTCAGAAACATAATATTCAGGCCCTGCTGAAAGATAGC
ATTGTTCAGCTGTGTACCGCACGTCCGGAACGTCCGATGGCATTTCTGCGCGAATA
TTTTGAACGTCTGGAAAAAGAAGAAGCCAAA
Sequence of CFP:
ATGGTGAGCAAGGGCGAGGAGCTGTTCACCGGGGTGGTGCCCATCCTGGTCGAG
CTGGACGGCGACGTAAACGGCCACAAGTTCAGCGTGTCCGGCGAGGGCGAGGGC
GATGCCACCTACGGCAAGCTGACCCTGAAGTTCATCTGCACCACCGGCAAGCTGC
CCGTGCCCTGGCCCACCCTCGTGACCACCCTGTCCTGGGGCGTGCAGTGCTTCGC
CCGCTACCCCGACCACATGAAGCAGCACGACTTCTTCAAGTCCGCCATGCCCGAA
GGCTACGTCCAGGAGCGCACCATCTTCTTCAAGGACGACGGCAACTACAAGACC
CGCGCCGAGGTGAAGTTCGAGGGCGACACCCTGGTGAACCGCATCGAGCTGAAG
GGCATCGACTTCAAGGAGGACGGCAACATCCTGGGGCACAAGCTGGAGTACAAC
TACTTTAGCGACAACGTCTATATCACCGCCGACAAGCAGAAGAACGGCATCAAGG
CCAACTTCAAGATCCGCCACAACATCGAGGACGGCGGCGTGCAGCTCGCCGACC
ACTACCAGCAGAACACCCCCATCGGCGACGGCCCCGTGCTGCTGCCCGACAACC
ACTACCTGAGCACCCAGTCCAAGCTGAGCAAAGACCCCAACGAGAAGCGCGATC
ACATGGTCCTGCTGGAGTTCGTGACCGCCGCCGGGATCACTCTCGGCATGGACGA
GCTGTACAAGTAA
Sequence of YFP:
TCTAAAGGTGAAGAATTATTCACTGGTGTTGTCCCAATTTTGGTTGAATTAGATGG
TGATGTTAATGGTCACAAATTTTCTGTCTCCGGTGAAGGTGAAGGTGATGCTACTT
ACGGTAAATTGACCTTAAAATTGATTTGTACTACTGGTAAATTGCCAGTTCCATGG
CCAACCTTAGTCACTACTTTAGGTTATGGTTTGCAATGTTTTGCTAGATACCCAGAT
CATATGAAACAACATGACTTTTTCAAGTCTGCCATGCCAGAAGGTTATGTTCAAGA
AAGAACTATTTTTTTCAAAGATGACGGTAACTACAAGACCAGAGCTGAAGTCAAG
TTTGAAGGTGATACCTTAGTTAATAGAATCGAATTAAAAGGTATTGATTTTAAAGA
AGGTGGTAACATTTTAGGTCACAAATTGGAATACAACTATAACTCTCACAATGTTT
ACATCACTGCTGACAAACAAAAGAATGGTATCAAAGCTAACTTCAAAATTAGACA
CAACATTGAAGATGGTGGTGTTCAATTAGCTGACCATTATCAACAAAATACTCCAA
TTGGTGATGGTCCAGTCTTGTTACCAGACAACCATTACTTATCCTATCAATCTGCCT
TATCCAAAGATCCAAACGAAAAGAGAGATCACATGGTCTTGTTAGAATTTGTTACT
GCTGCTGGTATTACCCATGGTATGGATGAATTGTACAAATAA

Sequence of MenD-MenH fusion:
ATGGCTAGCATGACTGGTGGACAGCAAATGGGTCGCGGATCCGAATTCGAGCTCA
TGTCTTCTCTGGTGCCACGCGGTTCTGGATCCTCAGTAAGCGCATTTAACCGACGC
TGGGCGGCGGTCATTCTGGAAGCATTAACGCGTCACGGCGTCAGACACATCTGTA
TCGCCCCAGGCTCGCGTTCTACACCGTTAACGTTAGCGGCGGCGGAGAATTCCGC
ATTCATTCACCACACCCATTTCGATGAGCGTGGGTTGGGGCATCTGGCGCTGGGG
CTGGCGAAAGTCAGCAAGCAGCCGGTGGCGGTGATTGTGACCTCCGGCACGGCG
GTGGCAAATCTCTATCCGGCACTGATTGAAGCCGGGTTAACCGGAGAAAAACTGA
TTCTCTTAACCGCCGATCGCCCGCCGGAGCTAATTGACTGCGGCGCGAATCAGGC
AATTCGCCAGCCGGGAATGTTCGCCTCTCACCCCACGCACAGTATTTCATTGCCGC
GCCCGACCCAGGATATCCCCGCACGTTGGCTGGTTTCTACCATCGACCACGCTCTC
GGTACGCTTCATGCGGGGGGAGTCCATATCAACTGCCCGTTTGCTGAACCGCTGTA
TGGCGAAATGGACGATACCGGGCTTAGCTGGCAACAGCGTCTGGGTGACTGGTG
GCAGGACGACAAACCGTGGCTGCGTGAAGCGCCTCGTCTGGAAAGTGAAAAAC
AGCGCGACTGGTTCTTCTGGCGACAAAAGCGCGGCGTGGTGGTTGCCGGGCGCA
TGAGTGCGGAAGAGGGCAAAAAAGTTGCCCTGTGGGCGCAAACTCTTGGCTGGC
CGCTGATTGGCGATGTGCTGTCACAAACCGGGCAGCCGCTGCCGTGTGCCGATCT
TTGGTTAGGCAATGCCAAAGCGACCAGCGAGCTGCAGCAGGCGCAAATTGTGGT
GCAACTGGGAAGCAGCCTGACGGGGAAACGGCTCCTGCAATGGCAGGCAAGCTG
TGAACCAGAAGAGTACTGGATTGTTGATGACATTGAAGGGCGACTTGATCCGGCA
CACCATCGCGGACGTCGCTTAATTGCCAATATTGCCGACTGGCTGGAGCTGCATCC
GGCAGAAAAACGCCAGCCCTGGTGCGTTGAAATCCCGCGCCTGGCGGAACAGGC
AATGCAGGCGGTTATTGCCCGCCGTGATGCGTTTGGCGAAGCGCAACTGGCGCAT
CGCATCTGCGACTATCTGCCTGAACAGGGGCAATTGTTTGTTGGTAACAGCCTGGT
GGTACGTCTGATTGATGCGCTTTCGCAACTTCCGGCAGGTTACCCGGTGTACAGCA
ACCGTGGGGCCAGCGGTATCGACGGGCTGCTTTCGACCGCCGCCGGCGTTCAGCG
GGCAAGCGGCAAACCGACGCTGGCGATTGTGGGCGATCTCTCCGCACTTTACGAT
CTCAACGCGCTGGCGTTATTGCGTCAGGTTTCTGCGCCGCTGGTATTAATTGTGGT
GAACAACAACGGCGGGCAAATTTTCTCGCTGTTGCCAACGCCGCAAAGCGAGCG
TGAGCGTTTCTATCTGATGCCGCAAAACGTCCATTTTGAGCACGCCGCCGCGATGT
TCGAGCTGAAATATCATCGTCCGCAAAACTGGCAGGAACTTGAAACGGCATTTGC
CGACGCCTGGCGCACGCCAACCACCACGGTGATTGAAATGGTGGTTAACGACAC
CGATGGTGCGCAAACGCTCCAGCAACTTCTGGCGCAGGTAAGCCATTTAGTCGAC
AAGCTTGGTGGCGGCGGCGGATCAATCCTGCACGCGCAGGCAAAACACGGAAAA
CCAGGTTTACCCTGGCTGGTGTTTTTGCACGGTTTTTCCGGCGATTGCCACGAATG
GCAAGAAGTGGGCGAGGCGTTTGCCGACTACTCACGGTTGTATGTTGATCTCCCA
GGTCACGGTGGTTCGGCGGCGATTAGCGTCGATGGATTTGATGATGTCACCGACTT
ACTGCGTAAAACCTTGGTTAGTTACAACATCCTTGACTTCTGGCTGGTGGGGTACT
CGCTTGGTGGACGGGTGGCGATGATGGCGGCTTGCCAGGGGCTGGCGGGGCTTT
GTGGGGTTATTGTCGAAGGCGGGCATCCGGGGCTGCAAAATGCTGAACAACGTGC
GGAACGTCAGCGTTCCGATCGCCAATGGGTGCAGCGTTTTCTCACAGAACCGTTA
ACGGCGGTATTTGCCGACTGGTATCAACAGCCTGTTTTTGCCTCACTCAATGACGA
TCAACGCCGGGAGCTGGTGGCGCTGCGCAGCAACAATAATGGCGCAACGCTTGC
CGCCATGCTGGAGGCGACTTCTCTCGCCGTCCAGCCTGATTTACGTGCTAACCTTA
GCGCCCGCACATTTGCGTTTTATTATTTATGTGGTGAACGTGACAGCAAATTCCGC
GCCCTGGCGGCGGAACTGGCTGCCGACTGCCATGTCATTCCTCGCGCCGGACATA

ACGCGCATCGGGAAAATCCCGCTGGCGTAATCGCAAGTCTGGCGCAGATCTTGCG
TTTCCTCGAGCACCACCACCACCACCACTGA

Supplementary Table 4. Primers used in this study

Primers	Sequence 5'-3' (restriction sites are underlined)
MenD-F	GGATCCTCAGTAAGCGCATTTAACCGACGCTGGGC
MenD-R	AAGCTTGGGAGCCTAAATGGCTTACCTGCGCCAGAA
MenH-F	GGATCCATCCTGCACGCGCAGGCAAAACACGGAAAA
MenH-R	AAGCTTGGGAGCCGAAACGCAAGATCTGCGCCAGACT
MenD$_{RA2}$-1-F	GGAAGCGACGGAAGGCTGTTAGCTCGAGGATCCGGCTGCTAACAAAGCC
MenD$_{RA2}$-1-R	AGAGAGTCAATTCAGGGTGGTGAATGTGAAACCAGTAACGTTATACGAT
MenD$_{RA2}$-2-F	ATCGTATAACGTTACTGGTTTCACATTCACCACCCTGAATTGACTCTCT
MenD$_{RA2}$-2-R	CAGGCAGATAGTCGCAGATGCGATGCGCCAGTTGCGCTTCGCCAAACGC
MenD$_{RA2}$-3-F	GCGTTTGGCGAAGCGCAACTGGCGCATCGCATCTGCGACTATCTGCCTG
MenD$_{RA2}$-31-R	TTGCTCCAGTCCACAGCCACCGCCTCCTGAACCTCCGCCTCCAGATCCACCGCCtCCGCAACCTTCGGTTGCTTCTTT
MenD$_{RA2}$-32-R	CTAACAGCCTTCCGTCGCTTCCTTAATGATCTGGTCGGCCAGTTGGTTCGCGTATTGCTCCAGTCCACAGCCACCGCC
MenD$_{RA2}$-33-R	GGCTTTGTTAGCAGCCGGATCCTCGAGCTAACAGCCTTCCGTCGCTTCC
82-1-F	TGGTGCCGCGCGGCAGCCATATGCAAACGGAACACGTCATTTTATTGA
82-1-R	CCACCACCTGAACCACCACCACCTTTAAGCTGGGTAAATGCAGATA
82-2-F	TATCTGCATTTACCCAGCTTAAAGGTGGTGGTGGTTCAGGTGGTGG
82-2-R	TATAGGGCGAATTGGAGCTCTTAGCAACCTTCGGTTGCTTCTTTGA
82-3-F	AAGCAACCGAAGGTTGCTAAGAGCTCCAATTCGCCCTATAGTGAG
82-3-R	CGCTGCGAATCGGGAGCGGCGATACCGTAAAGCACGAGGAAGCGGTCAGC
82-4-F	CTGACCGCTTCCTCGTGCTTTACGGTATCGCCGCTCCCGATTCGCAGCG
82-4-R	TCAATAAAATGACGTGTTCCGTTTGCATATGGCTGCCGCGCGGCACCA
1532-1-F	ATGACCGTGTGTGCGAAAAACATGTGCATCTGACCCGTG
1532-1-R	CCACCACCTGAACCACCACCACCCGACACCGCTGCCAGTTTTTATCG
1532-2-F	CGATAAAAACTGGCAGCGGTGTCGGGTGGTGGTGGTTCAGGTGGTGG
1532-2-R	TCGGTTTCATGGTTAATTCCTCCTTTATTTGGCTTCTTCTTTTTCCAG
1532-3-F	CTGGAAAAAGAAGAAGCCAAATAAAGGAGGAATTAACCATGAAACCGA
1532-3-R	GCTCAGCCAGGTCTGCACTGCCACGATGGCCGGCACGATC
1532-4-F	GATCGTGCCGGCCATCGTGGCAGTGCAGACCTGGCTGAGC
1532-4-R	CACGGGTCAGATGCACATGTTTTTCGCACACACGGTCAT

81f-1-F	GGAGGTTCAGGTGGAGGTGGATCTATGGTGAGCAAGGGCGAGGAGCTG
81f-11-F	ATCTGCATTTACCCAGCTTAAAGGAGGTGGAGGTTCAGGTGGAGGTGGAT
81f-1-R	GACTCACTATAGGGCGAATTGGAGCTCCTACTCCTTGTACAGCTCGTCCATG
81f-2-F	CATGGACGAGCTGTACAAGGAGTAGGAGCTCCAATTCGCCCTATAGTGAGTC
81f-2-R	CCAAAAATAATAACCTTTCCCGGTGCAGAAGTTAAGAACGGTAATGAC
81f-3-F	GTCATTACCGTTCTTAACTTCTGCACCGGGAAAGGTTATTATTTTTGG
81f-3-R	CAGCTCCTCGCCCTTGCTCACCATAGATCCACCTCCACCTGAACCTCC
82f-1-R	TTCCAGACCACAtCCtCCaCCaCCAGACTCCTTGTACAGCTCGTCCATG
82f-2-F	CATGGACGAGCTGTACAAGGAGTCTGGTGGTGGAGGATGTGGTCTGGAA
82f-2-R	CCAAAAATAATAACCTTTCCCGGTGCAGAAGTTAAGAACGGTAATGAC
82f-3-F	GTCATTACCGTTCTTAACTTCTGCACCGGGAAAGGTTATTATTTTTGG
82f-3-R	CAGCTCCTCGCCCTTGCTCACCATAGATCCACCTCCACCTGAACCTCC
1532f-1-F	GTGGTTCAGGTGGaGGtGGaTCTTCTAAAGGTGAAGAATTATTCACTGGTGTTG
1532f-1-R	GCTTCCACAACCCCCACCGCCTTTGTACAATTCATCCATACCATGGGTA
1532f-2-F	TACCCATGGTATGGATGAATTGTACAAAGGcGGTGGgGGTTGTGGaAGC
1532f-2-R	GATTATTCATGAGTATTACCTCCTTTATTTGGCTTCTTCTTTTTCCAGACG
1532f-3-F	CGTCTGGAAAAAGAAGAAGCCAAATAAAGGAGGTAATACTCATGAATAATC
1532f-3-R	AGGCCCCAAGGGGTTATGCTAGTTATTGCTCAGCGGTGGCAGCAGCCTA
1532f-4-F	TAGGCTGCTGCCACCGCTGAGCAATAACTAGCATAACCCCTTGGGGCCT
1532f-4-R	CAACACCAGTGAATAATTCTTCACCTTTAGAAGAtCCaCCtCCACCTGAACCAC
crtE_left-F	CGCATTGTTATCTTTCGCTTTTGAA
RI-crtE R	CCACCACCTGAACCACCACCACCGTTTTGCCTGAAAGCGATGTAAT
crtE-RI F	ATTACATCGCTTTCAGGCAAAACGGTGGTGGTGGTTCAGGTGGTGG
GPM RI R	ATCAAATCATTCATTCTTCAGACTTATTTGGCTTCTTCTTTTTCCA

RI-GPM F	TGGAAAAAGAAGAAGCCAAATAAGTCTGAAGAATGAATGATTTGAT
crtE_RI-terminator-R	ATTAAGGGTTGTCGACCTGCAGCTATTCGAACTGCCCATTC
crtE_hygR-F	GAATGGGCAGTTCGAATAGCTGCAGGTCGACAACCCTTAAT
crtE_hygR-R	CATAAAAAAAGAGAATCTTTCCACTAGTGGATCTGATATC
crtE_right-F	GATATCAGATCCACTAGTGGAAAGATTCTCTTTTTTTATG
crtE_right-R	TCTTCCATCAGATTTGGTATTGGTA
idi left F	ACAAAGAGCCACTGAAAAAATAA
RIAD idi R	CCACCACCTGAACCACCACCACCTAGCATTCTATGAATTTGCCTGT
idi RIAD F	ACAGGCAAATTCATAGAATGCTAGGTGGTGGTGGTTCAGGTGGTGG
Ter RIAD R	GTAGCCTATATTATTGACGCGTTGTTAGCAACCTTCGGTTGCTTC
RIAD Ter F	GAAGCAACCGAAGGTTGCTAACAACGCGTCAATAATATAGGCTAC
G418 Ter R	TAAGGGTTGTCGACCTGCAGCGTGAAGAGAAAAAAAAAATGTGAAC
TER G418 F	GTTCACATTTTTTTTTCTCTTCACGCTGCAGGTCGACAACCCTTA
MRP G418 R	GAATATAATCAAGGGAAATTGACCACTAGTGGATCTGATATCACC
G418 MRP F	GGTGATATCAGATCCACTAGTGGTCAATTTCCCTTGATTATATTC
MRP R	ATGTAGTGAAGTTTCTAAAC
1530-F	GTATAAGAAGGAGATATACATAGGAGGATTACACTATGCCGCGGTA
1530-R	CGCAGCAGCGGTTTCTTTACCAGACTCGAGTTAGCTCTCACCACGCCATA
15322-1-F	GTTAAGTATAAGAAGGAGATATACATATGCAAACGGAACACGTCATT
15322-1-R	CGGCATAGTGTAATCCTCCTTTAGCAACCTTCGGTTGCT
15322-2-F	AGCAACCGAAGGTTGCTAAAGGAGGATTACACTATGCCG
15322-2-R	CAGCGGTTTCTTTACCAGACTCGAGTTAGCTCTCACCACGCCATAAGCG
15301-1-F	GCTGTCGAAATGGTTCATGCGGCTAGCCTGATCCTGGATGACATGC
15301-1-R	TTGCATGCCTCCGCCGCTGCCGCCCGACACCGCTGCCAGTTTTTTATCG
15301-2-F	TGTCGGGCGGCAGCGGCGGAGGCATGCAAACGGAACACGTCATTTTATTG
15301-2-R	GGTTTCATGGTTAATTCCTCCTTTATTTAAGCTGGGTAAATGCAG
15301-3-F	CTGCATTTACCCAGCTTAAATAAAGGAGGAATTAACCATGAAACC
15301-3-R	CAGGTGTTCATCTTCGATGTAGCTAGCAACTTTAGAATACACGCTACGCC
15302-1-F	TTGTGAGCGGATAACAATTCCCCTCTAGACCATCTTAGTATATTAGTTA
15302-1-R	TCATGCCTCCGCCGCTGCCGCCTTTAAGCTGGGTAAATGCAG
15302-2-F	CAGCTTAAAGGCGGCAGCGGCGGAGGCATGACCGTGTGTGCGAAAAAAC
15302-2-R	GCGGCCTGCATGGGCGTCATCCACGATATCACGTGCAATATTCGTCAGCT

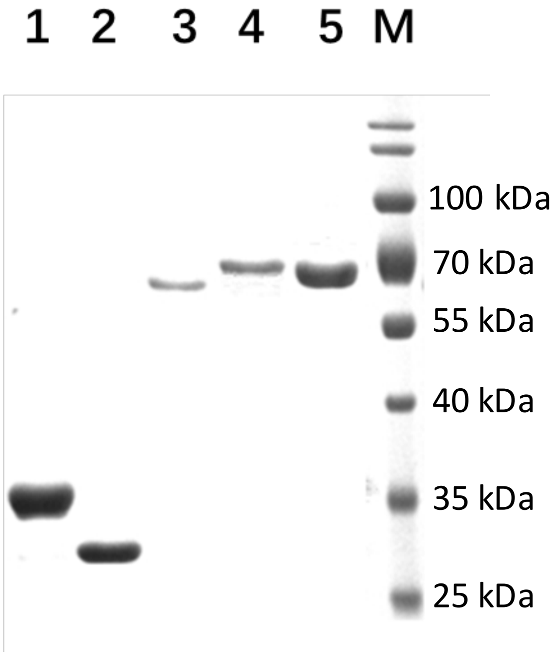

Supplementary Figure 1. Purified $MenH_{RD}$ (lane 1), $MenH_{RA}$ (lane 2), $MenD_{RA}$ (lane3), $MenD_{RA2}$ (lane 4) and $MenD_{RD}$ (lane 5) analyzed by SDS-PAGE. M, marker. Source data are provided as a Source Data file.

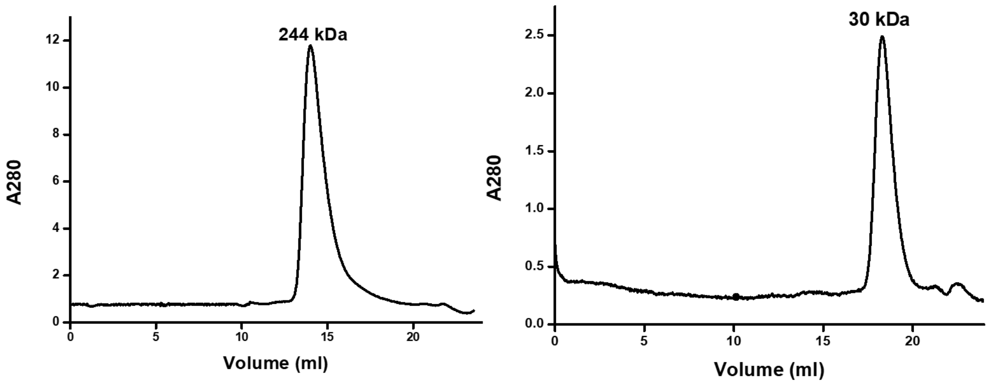

Supplementary Figure 2. Purified MenD (left) elutes as a tetramer, and MenH (right) as a monomer on Superdex 200 10/300 GL in SEC. Source data are provided as a Source Data file.

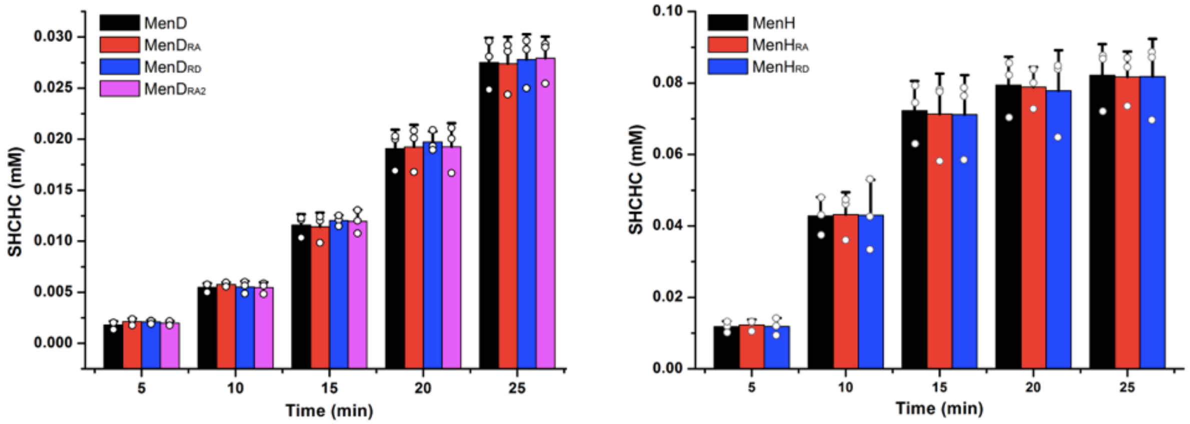

Supplementary Figure 3. Peptide tagging did not change the activity of MenD (left) and MenH (right). In the left, black column: MenD; red column: $MenD_{RA}$; blue column: $MenD_{RD}$; pink column: $MenD_{RA2}$. In the right, black column: MenH; red column: $MenH_{RA}$; blue column: $MenH_{RD}$. Error bars indicate the standard deviations of three biological replicates. Source data are provided as a Source Data file.

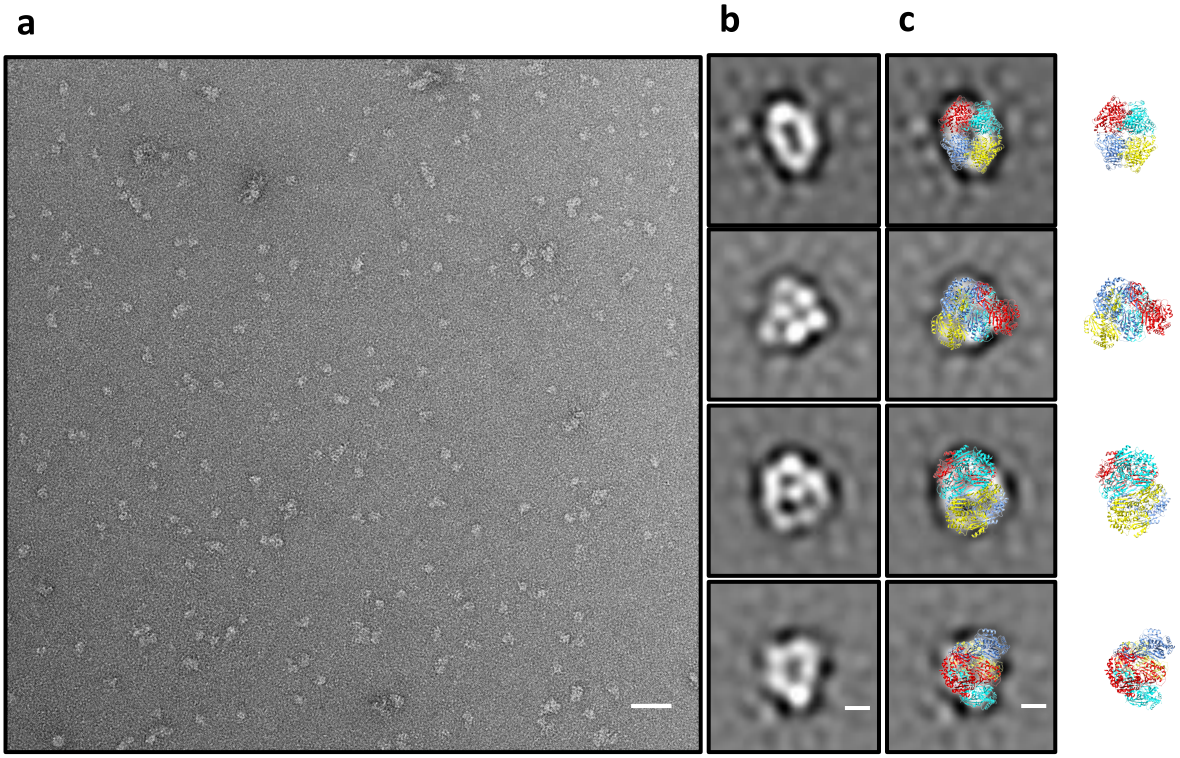

Supplementary Figure 4. TEM images of the Assembly B. **a** Raw electron micrograph. Scale bar, 50 nm. **b** 2D classification performed using RELION 2.0. Scale bar, 5 nm. **c** Superimposition of the crystal structure of MenD tetramer (PDB ID 2JLA) to the 2D classes averages. Scale bar, 5 nm. Source data are provided as a Source Data file.

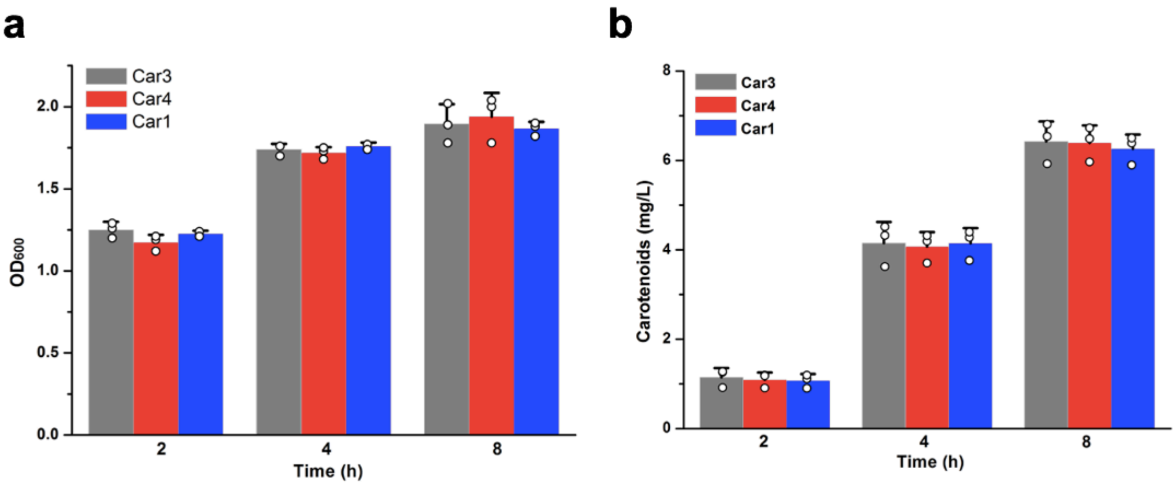

Supplementary Figure 5. Strains Car3 and Car4 have identical carotenoids production rates and growth rates as the untagged control Car1 in shake-flask fermentation. **a** The growth of strains. **b** The accumulation of carotenoids. Grey column: Car3; red column: Car4; blue column: Car1. Error bars indicate the standard deviations of three biological replicates. Source data are provided as a Source Data file.

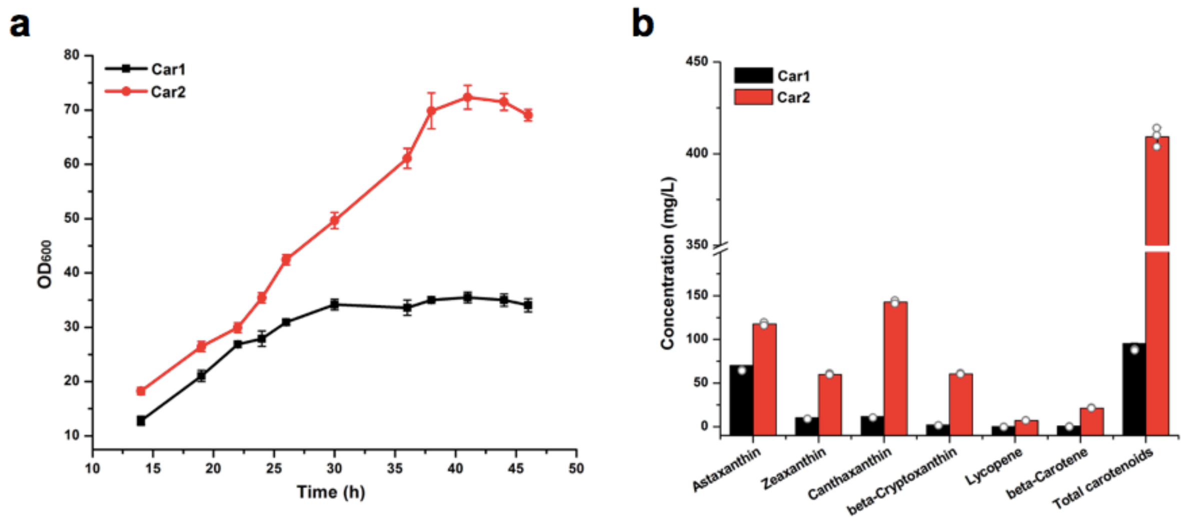

Supplementary Figure 6. Independent fed-batch fermentations showing the same trend of carotenoid production. **a** Comparison of the growth curve and the yield of overall carotenoids of Car2 and Car1 in fed-batch fermentation. Black line: Car1; red line: Car2. **b** Comparison of the product of main carotenoids. Black column: Car1; red column: Car2. Error bars indicate the standard deviations of three replicates. Source data are provided as a Source Data file.

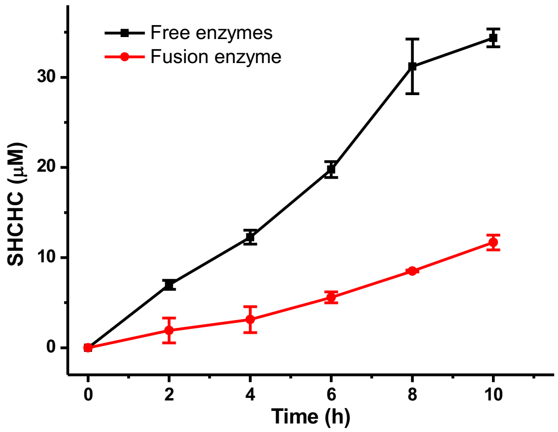

Supplementary Figure 7. Comparison of enzyme activity of the MenD-MenH fusion enzyme and free MenD and MenH in 1:1 ratio, showing the enzyme fusion has significantly reduced activity. The activity of converting isochorismate to SHCHC by MenD and MenH sequentially was compared, and the generation rate of SHCHC was recorded. Black line: Free enzymes; red line: Fusion enzyme. Error bars indicate the standard deviations of two replicates. Source data are provided as a Source Data file.

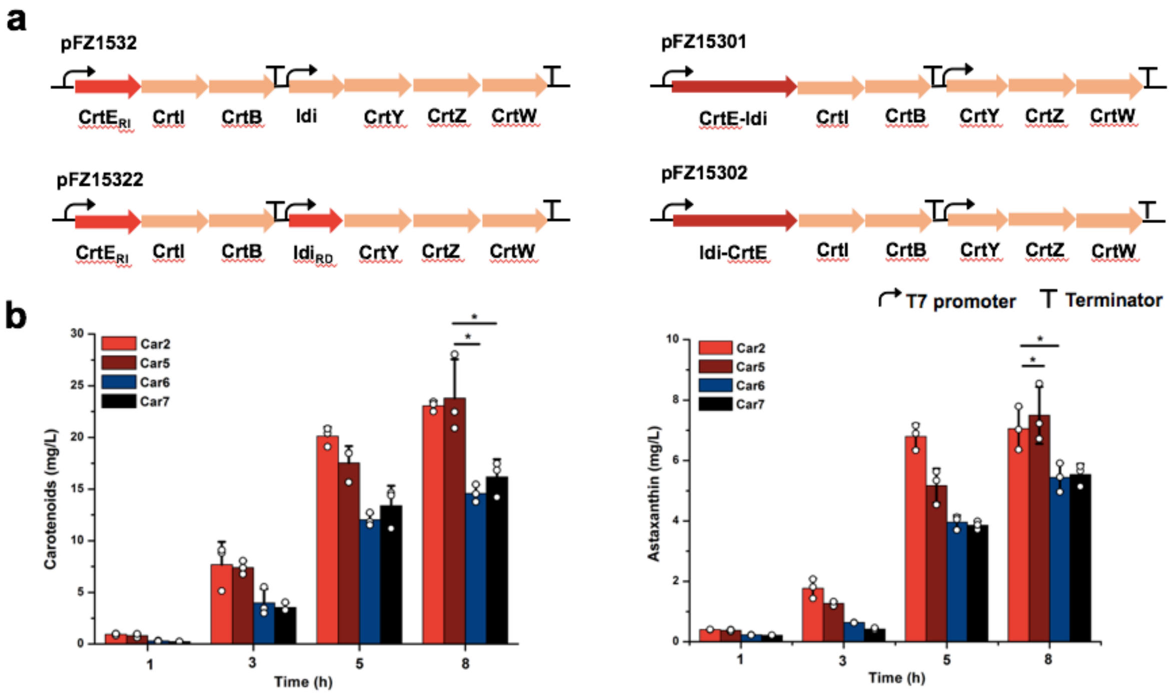

Supplementary Figure 8. Comparison of the assembly and fusion strains, showing that strains with enzyme assembly have a higher product titer during shake-flask fermentation. **a** Construction of Car5-7 strains. Car5 is an assembly strain with both Idi and CrtE under T7 promoter control in the same plasmid. Car6 and Car7 are fusion strains containing CrtE-Idi or Idi-CrtE fusion enzymes respectively. Car2 contains plasmids pMH1, pFZ82, pFZ1532. Car5 contains plasmids pMH1, pFZ81, pFZ15322. Car6 contains plasmids pMH1, pFZ81, pFZ15301. Car7 contains plasmids pMH1, pFZ81, pFZ15302. **b** The comparison of astaxanthin and total carotenoids production titer between the four strains. The two assembly strains Car2 and Car5 produce significantly higher (about 40% higher) product titer than the two fusion trains Car6 and Car7. Red column: Car2; wine column: Car5; blue column: Car6; black column: Car7. *$P<0.05$ (Student's t-test: three-tailed, three-sample equal variance). Error bars indicate the standard deviations of three biological replicates. Source data are provided as a Source Data file.

Supplementary References

1. Zhu, F. et al. *In vitro* reconstitution of mevalonate pathway and targeted engineering of farnesene overproduction in *Escherichia coli*. *Biotechnol. Bioeng.* **111**, 1396-1405 (2014).
2. Ma, T. et al. Genome mining of astaxanthin biosynthetic genes from *Sphingomonas* sp. ATCC 55669 for heterologous overproduction in *Escherichia coli*. *Biotechnol. J.* **11**, 228-237 (2016).
3. Ma, T. et al. Lipid engineering combined with systematic metabolic engineering of *Saccharomyces cerevisiae* for high-yield production of lycopene. *Metab. Eng.* **52**, 134-142 (2019).
